# LASSO logistic regression and cluster analysis in predicting adherence and drug patterns among new users of monotherapy for antihypertensive drugs

**DOI:** 10.1097/HJH.0000000000004084

**Published:** 2025-06-20

**Authors:** Xuechun Li, Mutiara Djayanis Tia, Jens H.J. Bos, Catharina C.M. Schuiling-Veninga, Eelko Hak, Sumaira Mubarik

**Affiliations:** PharmacoTherapy, -Epidemiology and -Economics, Groningen Research Institute of Pharmacy, University of Groningen, Groningen, The Netherlands

**Keywords:** adherence, clustering, drug pattern, Least Absolute Shrinkage and Selection Operator, logistic regression, risk factors

## Abstract

**Objective:**

Real-world long-term adherence and drug utilization patterns are essential for hypertension management. However, the evidence was unclear. We aimed to construct adherence and drug patterns risk prediction models.

**Methods:**

We designed a retrospective inception cohort study using pharmacy records from the University of Groningen IADB.nl dispensing database. Exposures were antihypertensive drug monotherapy including angiotensin-converting enzyme inhibitors, angiotensin II receptor blockers, beta-blockers, calcium channel blockers, and thiazides. Primary outcomes included adherence rates and drug utilization patterns. Cluster analysis was performed to categorize patients with similar risk factors. Least Absolute Shrinkage and Selection Operator logistic regression was then employed to construct prediction models.

**Results:**

The adherence rate in the 1^st^ year to the 10^th^ year increased from 86.4% to 92.9%. Most monotherapies, middle and older age, with initial diabetes, asthma/ chronic-obstructive-pulmonary-disease drug, and without psycholeptics drug, first exposure prescription after 2000 promoted high adherence. Thiazides and being male were harmful to continuation and helpful to discontinuation, switching and adding on. Middle and older age promoted switching and adding on but impeded discontinuation. High adherence contributed to continuation, switching and adding on but impeded discontinuation. Thiazides were better for the short term use to achieve high adherence while calcium channel blockers in both short term and long term were better to achieve high adherence than beta-blockers.

**Conclusions:**

The results highlighted key factors influencing medication adherence and treatment changes, emphasizing the need for personalized approaches to optimize patient care. Enhancing adherence and provide specific monotherapy for short term and long term plan were beneficial to better personalized treatment.

## INTRODUCTION

Hypertension is a significant risk factor for cardiovascular disease (CVD) [1]. In primary prevention, traditional antihypertensive monotherapy has been the mainstream hypertension treatment, though it has some limitations [2]. Also in the Netherlands, cardiovascular risk management involves starting with a low-dose of a single drug [3].

Inadequate adherence is a widely acknowledged factor contributing to the ineffective management of blood pressure in individuals with hypertension [4]. In numerous countries worldwide, and despite the prevalent utilization of monotherapy, a significant proportion of hypertensive patients continue to experience uncontrolled blood pressure levels [2]. Interestingly, in studies from the UK and the Netherlands, 80–91% of patients on such monotherapy showed an adherence rate ≥0.8 [5,6].

In addition to adherence, prescription patterns may also reflect the impact of the current guidelines. After starting antihypertensive drug monotherapy, for instance, patients may need to discontinue treatment or switch to or add on another antihypertensive drug. However, not much is known about such drug patterns, notably not in the long term.

Risk factors including comorbidities, unhealthy lifestyles, and health insurance may influence the adherence of patients who are using antihypertensive drug monotherapy [7,8]. Although common, only a few studies have explored whether concomitant medications affect adherence or lead to other drug patterns.

Regarding patients’ blood pressure management and medication patterns, predicting adherence or drug utilization patterns according to risk factors is essential. Therefore, we constructed risk prediction models for adherence and drug patterns employing clustering methods and LASSO (Least Absolute Shrinkage and Selection Operator) regression. As part of unsupervised learning [9,10], clustering analysis could divide patients into several groups with similar distinct risk factors. Such clustering techniques utilize a distance or similarity metric to measure the proximity between data points, whereas LASSO logistic regression is a regularization method, aids in feature selection and includes the most significant factors in the model [11]. LASSO is especially beneficial for high-dimensional datasets, as it enhances interpretability and reduces model complexity by effectively handling multicollinearity. Both clustering methods and LASSO regression are used many times in medicine daily practice [12–15]. With these two methods, we can provide more detailed information for pharmacies to develop personalized drug interventions.

## MATERIAL AND METHODS

### Design and setting

We designed a longitudinal inception cohort study of adult starters with any of the advised antihypertensive drug monotherapies (see exposures) for primary prevention of CVD. Data selection has previously been described [16]. In brief, data were extracted from the University of Groningen IADB.nl dispensing database from 1 January 1996 to 31 December 2020 [17,18]. A patient's index date was the date of the first prescription for antihypertensive drug monotherapy. Patients who had any other acute cardiovascular drug therapy, or prescriptions for chronic stable heart failure, migraine, adrenal disease, hyperparathyroidism or thyroid problems drugs in the 2 years before or within 90 days after the index date were excluded to ensure the inclusion of only those free from CVD and secondary hypertension; the end of the study period was defined as 31 December 2020. One follow-up period of 180 days represented half a year. The end of the follow-up time was defined as the end of the study period, or as a maximum of 3780 days (which represents 10 years) from the index date, the first date of acute cardiovascular drug therapy, the final date of the last prescription of exposure, or the date on which the patient changed their initial drug monotherapy, whichever came first.

Among the 33 427 adult starters with antihypertensive drug monotherapy, we selected three types of patients based on their length of follow up time or prescription records, to evaluate three main objectives: described the adherence in patients on original antihypertensive monotherapy exceeding 360 days (representing 1 year), 720 days (2 years), 1080 days (3 years), 1440 days (4 years), 1800 days (5 years), 2160 days (6 years), 2520 days (7 years), 2880 days (8 years), 3240 days (9 years), 3600 days (10 years); described the drug patterns in patients on original antihypertensive monotherapy exceeding 360 days; constructed adherence and drug patterns risk prediction models in patients on original antihypertensive monotherapy exceeding 360 days; evaluated the 3-year (1080 days) comparative risk among different antihypertensive monotherapies of experiencing high adherence in patients with all drug records exceeding 1080 days.

### Exposures

The Dutch guidelines [19] recommend any of the following five monotherapies for hypertension and CVD prevention: thiazides (anatomical therapeutical chemical code: C03AA), calcium channel blockers (CCBs: C08C, C08D, C08E), angiotensin converting enzyme inhibitors (ACEIs: C09A), angiotensin II receptor blockers (ARBs: C09C), and beta-blockers (BBs: C07A).

### Outcome

Primary outcomes included adherence rates, calculated as the proportion of days covered by antihypertensive monotherapy during follow-up from 0 to 1. The formula (1) was used in objective 1: to evaluate the consistency of adherence rates over the first ten years of treatment; in objective 2: to build the risk prediction models using the adherence rate in the 1^st^ year. While formula (2) was used in objective 3: to compare adherence rates among antihypertensive monotherapies over the 3-year follow up time. Binary adherence classified rates as high (≥0.8) or low (<0.8), based on earlier research [20].


(1) $$\frac{Total\;covereddaysin1st,2nd,\;3rd,4th\ldots 10thyear}{360\;days/years}$$

(2) $$\frac{Total\;covered\;days\;during\;follow-up\;period}{Total\;follow\;up\;period\;in\;days}$$

Secondary outcomes encompassed drug patterns, such as discontinuation, continuation, switch, and add-on. Discontinuation was defined as not receiving the initial prescription from the same class of the five classes of antihypertensive drug monotherapy for more than 180 days. Continuation was defined as continuing the baseline monotherapy from the index date to the end of follow up time, without drug discontinuation, without drug switch, and without drug add-on. A drug switch was defined as prescribing a new class of antihypertensive drug monotherapy or antihypertensive drug fixed-dose combinations within 180 days after discontinuation of a specific drug therapy (gap >180 days). Add-on was defined as prescribing a new drug class or antihypertensive drug fixed-dose combinations in addition to an existing drug class before discontinuation.

### Potential risk factors for high-adherence or drug patterns

We retrieved information on age at index date (18–39: young, 40–69: middle, ≥70: older) and sex (male, female) from the database. Initial drug use to treat comorbidities was defined as at least one prescription for the following diseases in the first 180 days from index date: diabetes (yes, no), rheumatoid arthritis (RA: yes, no), asthma/ chronic obstructive pulmonary disease (COPD: yes, no), antiepileptics (yes, no), antiparkinson (yes, no), psycholeptics (yes, no), psychoanaleptic (yes, no), addictive disorders (yes, no), and antineoplastic (yes, no). Further information was collected on calendar year of first prescription of exposure (1996–2000, 2000–2010, 2010–2020). Risk factor for discontinuation, continuation, switch, or add-on also contained adherence rate defined as high (adherence rate ≥80%) or low (adherence rate <80%).

### Statistical analysis

#### Objective 1

We used mean ± standard deviations (SDs) for continuous adherence and frequencies as proportions and percentages for high and low adherence as well as drug patterns rates. A significance level of α = 0.05 was set for the two-sided tests to determine statistical significance. Plots were generated to illustrate adherence and drug patterns.

#### Objective 2

##### Cluster analysis

Cluster analysis was conducted on potential risk factors for high adherence or drug patterns. The Gower distance calculation method [21] and partition around medoids clustering method [22] were utilized for this analysis. Patients were categorized into K groups with similar characteristics and comorbidity medication profiles. The size of K was determined using silhouette width plots [23]. The ‘cluster’ package in R was used for analysis.

##### Prediction model selection

We employed the glmnet and ‘caret’ packages to construct three penalized regression models: ridge, LASSO, and elastic net. The evaluation of these models utilized two metrics: Root Mean Square Error (RMSE) and R-squared. RMSE [24] gauged the accuracy of the regression model in predicting the response variable's value, while R-squared [25] measured how well the predictor variables explain the variation in the response variable. The model with the lowest RMSE and highest R-squared was considered the best and was subsequently compared with the logistic regression model.

For LASSO regression, we utilized a 7 : 3 data split and performed a 10-fold cross-validation to filter the variables and compute the area under the curve (AUC). Logistic regression's AUC and mean AUC were computed through simple cross-validation on the 7 : 3 data split and a 10-fold cross-validation. Ultimately, our analysis revealed that LASSO regression achieved the relatively highest AUC (see Table 1, Supplemental Digital Content).

##### LASSO logistic regression models of adherence and drug patterns

To investigate the independent effect of drug type on adherence outcomes (high/low) and drug patterns, we utilized LASSO logistic regression while controlling for the factors mentioned above. Additionally, LASSO logistic regression analysis was conducted within K-clustered groups of patients.

Before implementing LASSO logistic regression, associations were examined using Cramér's V [26] and mosaic plots [27], while multicollinearity was assessed with the vif function. Categorical independent variables were transformed into dummy variables using the model.matrix function. Subsequently, the dataset was divided into training and test sets at a ratio of 7 : 3. The LASSO model was then fitted to the training set and 10-fold cross-validation was utilized to determine the optimal lambda, which was subsequently employed to construct the predictive LASSO model applied to the test set. The model's performance was evaluated using sensitivity, specificity, and AUC. Finally, variables with at least one coefficient surviving LASSO selection within their categories were included in the final logistic regression model.

#### Objective 3

We drew 3-year Kaplan–Meier curve of probability of maintaining in low adherence for patients with antihypertensive drug monotherapy. We then facilitated Cox regression analysis of high adherence in patients with and without time-dependent effects, we compared the relative risk among monotherapy users before and after inverse probability weighting (IPW): a method to balance the baseline potential variables, in case to evaluate the baseline risk and overall risk.

### Sensitivity analysis

We performed logistic regression analysis in objective 2 patients and K-clustered groups of data without employing LASSO adjustment. In K-clustered groups, certain independent variables had only one level in some groups. Therefore, we chose to exclude these variables from the logistic regression model to ensure robustness and avoid issues related to model convergence. Furthermore, we performed the Hosmer-Lemeshow test and Nagelkerke *R*^2^ for both lasso and logistic regression model to evaluate the goodness-of-fit and how well the model explain the variation in the outcome. We also estimated time-dependent receiver operating characteristic curve, and reported the AUC, sensitivity, specificity, Nagelkerke's *R*^2^, and Harrell's *C*-index for the Cox regression analysis to assess model performance. R statistical software was used for both data preprocessing and analysis purposes.

## RESULTS

### Average adherence

**FIGURE 1:**
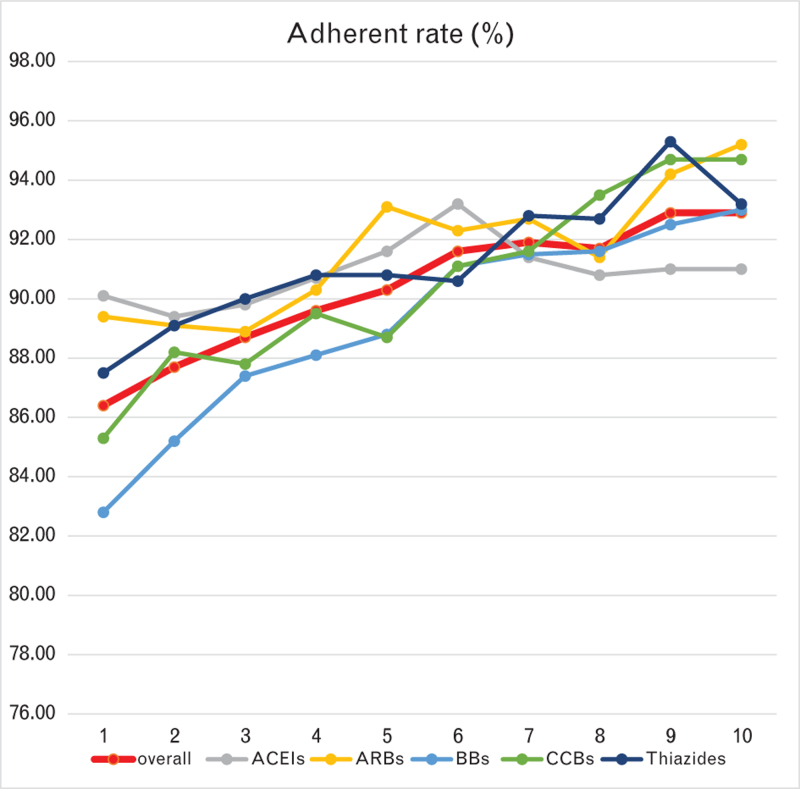
Adherence rate (adherence ≥ 0.8) from the 1^st^ to the 10^th^ year for patients who maintained antihypertensive monotherapy for more than 360 days (1 year),720 days(2 years),1080 days (3 years),1440 days (4 years),1800 days (5 years), 2160 days (6 years), 2520 days (7 years), 2880 days (8 years), 3240 days (9 years), 3600 days (10 years). ACEIs, angiotensin converting enzyme inhibitors; ARBs, angiotensin II receptor blockers; BBs, beta-blockers; CCBs, calcium channel blockers.

Among all 33 427 patients at baseline, the average adherence from the first year to the tenth year increased from 0.92 (0.14) to 0.94 (0.10), and the adherence rate increased from 86.4% to 92.9%, showing an upward trend. The average adherence and adherence rates to ACEIs and ARBs at 1^st^ year were the highest among other monotherapies while ARBs and CCBs were the highest at the 10^th^ year (see Table 2, Supplemental Digital Content and Fig. 1).

### Drug patterns

**FIGURE 2:**
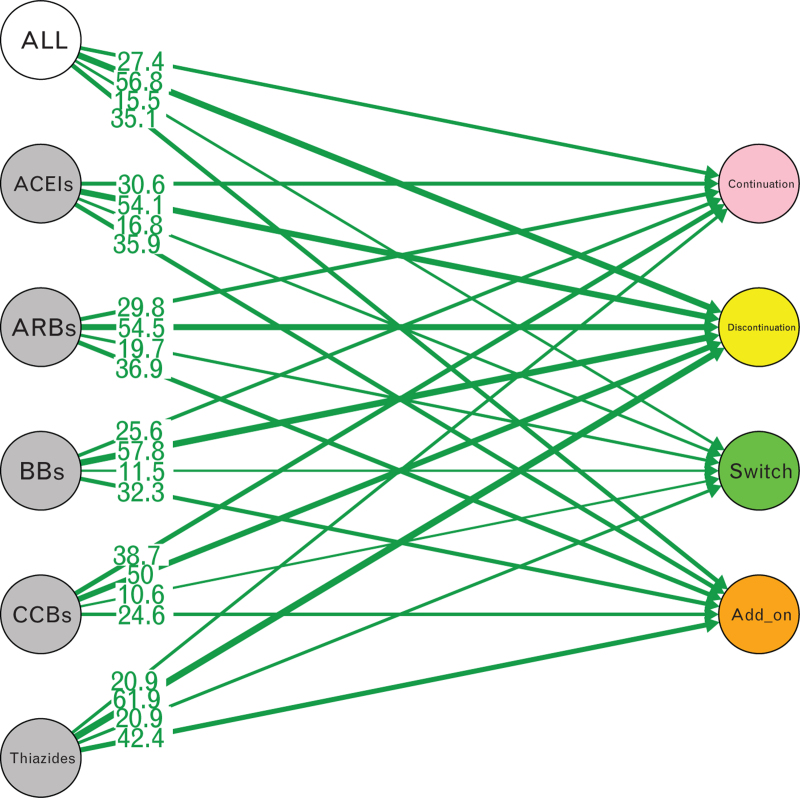
General drug patterns (%) of new users of antihypertensive drug monotherapies. ACEIs, angiotensin converting enzyme inhibitors; ARBs, angiotensin II receptor blockers; BBs, beta-blockers; CCBs, calcium channel blockers.

In general, CCB users were more likely to continue the original monotherapy, less likely to discontinue, and less likely to make changes. Thiazide users were the opposite. Thiazide users had the lowest continuation rate (20.9%), highest discontinuation rate (61.9%), switch rate (20.9%), and add-on rate (42.4%) of all monotherapy users, while CCB users showed the highest continuation rate (38.7%), lowest discontinuation rate (50.0%), switch rate (10.6%), and add on rate (24.6%) (see Table 3, Supplemental Digital Content and Fig. 2).In general, BB, CCB and Thiazide users switched to ACEIs and added on the ACEIs the most (see Fig. 3 ).

**FIGURE 3:**
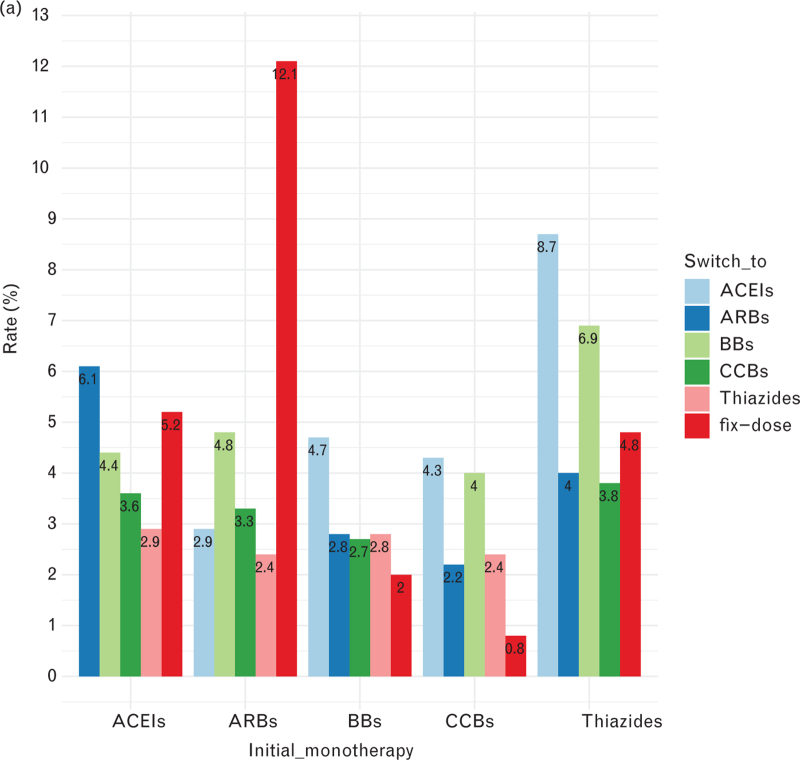
Five classes of drug users switch pattern and add-on patterns. (a) Switch patterns, (b) add on patterns. ACEIs, angiotensin converting enzyme inhibitors; ARBs, angiotensin II receptor blockers; BBs, beta-blockers; CCBs, calcium channel blockers.

**FIGURE 3 (Continued):**
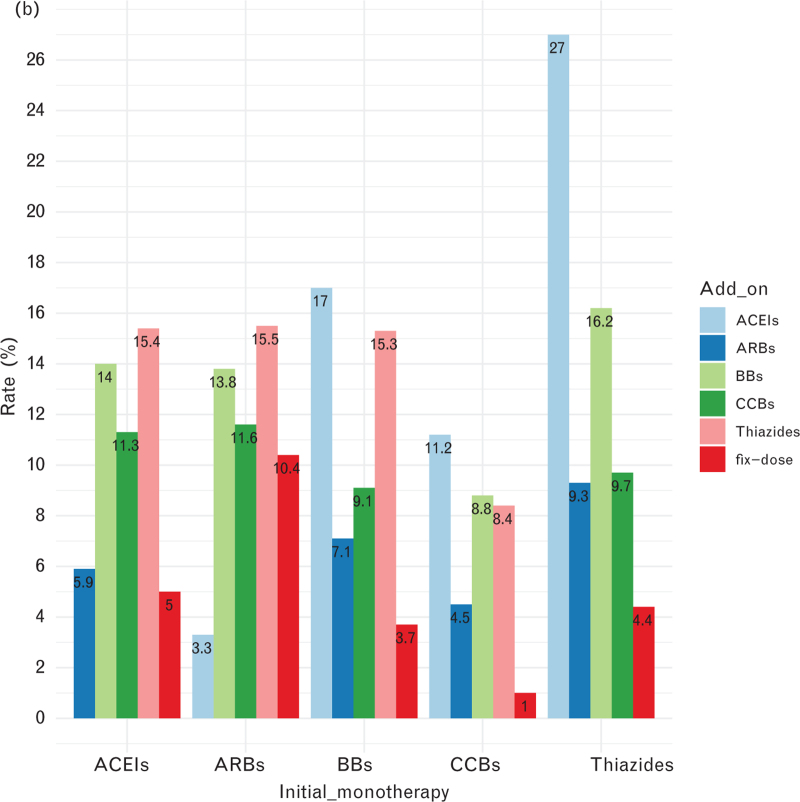
Five classes of drug users switch pattern and add-on patterns. (a) Switch patterns, (b) add on patterns. ACEIs, angiotensin converting enzyme inhibitors; ARBs, angiotensin II receptor blockers; BBs, beta-blockers; CCBs, calcium channel blockers.

### Cluster results

**FIGURE 4:**
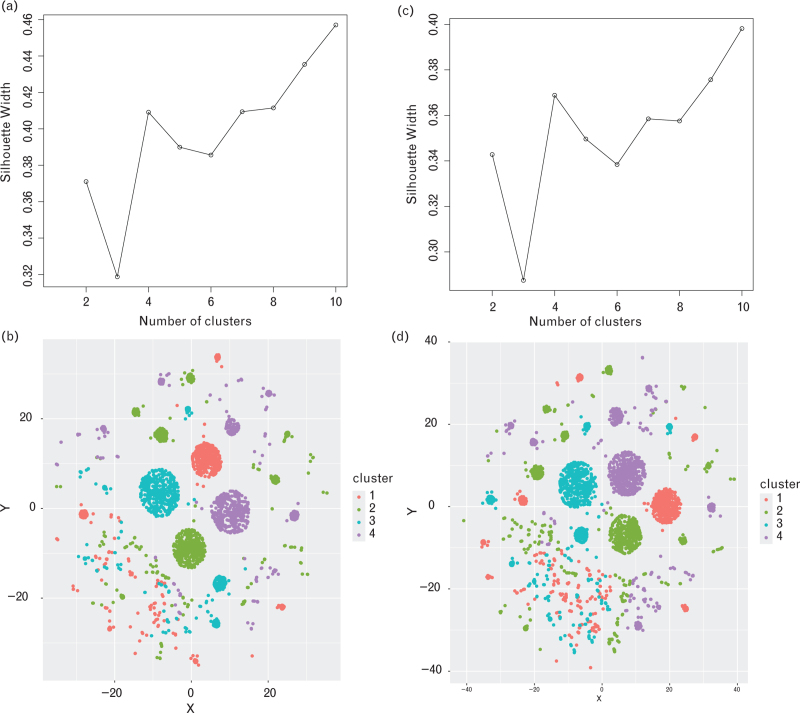
Silhouette score and clustering results for adherence and drug patterns as outcome. (a) Silhouette Score (outcome=adherence), (b) clustering result (outcome=adherence), (c) silhouette score (outcome = drug patterns), (d) clustering result (outcome = drug patterns).

Four clusters were formed based on similar baseline characteristics predicting adherence. After adding adherence as the adjustment variable, the number of clusters remained the same when drug patterns were the outcome (see Table 4, Supplemental Digital Content and Fig. 4).

### Adherence risk prediction model

In patients on original antihypertensive monotherapy exceeding 360 days (1 year), patients using ACEIs, ARBs, and thiazides, middle and older age, with initial diabetes drugs, asthma/COPD drugs, calendar year after 2000 were more likely to have high adherence, while initial psycholeptics drug users were the opposite. The same trends can be seen in all clusters, and CCB users also showed high adherence possibility in cluster 3 and 4 (see Table 5, Supplemental Digital Content).

### Drug pattern risk prediction model

Thiazides, being male, initial diabetes and psycholeptics drugs were harmful to continuation. CCBs, high adherence, middle age group, 2010–2020 calendar year were benefit to it. Thiazides harmed continuation in all clusters while high adherence was helpful to continuation across all clusters. Older age group was not beneficial to continuation in cluster 1 and 2 but was beneficial in cluster 3 and 4 (see Table 6, Supplemental Digital Content).

Thiazides, being male, initial asthma/COPD, drugs, antiparkinson drugs, psycholeptics drugs facilitated discontinuation. High adherence, middle and older age, calendar year after 2010 impeded discontinuation. Similar trends can be seen in all clusters (see Table 7, Supplemental Digital Content).

ACEIs, ARBs, CCBs, thiazides, high adherence, being male, middle and older age promoted switching while calendar year after 2010 was the opposite. Similar trends were in four clusters (see Table 8, Supplemental Digital Content).

The promoted and preventive factors to add on were partly same with switching, the difference was initial diabetes drugs was promoted factor, CCBs, antiparkinson drugs, psychoanaleptic drugs were preventive factors. Similar trends were in four clusters (see Table 9, Supplemental Digital Content).

### Relative risk of high adherence

The Figure 1, Supplemental Digital Content reflected transition from low adherence to high adherence within the first three years after index date, which indicated a decreasing probability of remaining in low adherence over time. The Figure 2, Supplemental Digital Content reflected the same transition among five classes of antihypertensive monotherapies,

After IPW adjustment, the Cox regression without time-dependent effects showed that, compared to BB users, ACEI, ARB and Thiazide users had an average lower likelihood of high adherence in the entire follow up time, CCB users had an average higher likelihood of high adherence. While when we added time-dependent effects, thiazide and CCB users both had higher likelihood of high adherence at baseline, the AUC at 1080 days, sensitivity, and specificity were lower compared to without time-dependent effects. Nagelkerke's *R*^2^ increased slightly, while Harrell's *C*-index decreased slightly. Compared to before IPW adjustment, almost all indices increased slightly after IPW adjustment (see Table 1 and Figure 2, Supplemental Digital Content).

**TABLE 1:** Cox regression analysis of high-adherence (14 628 patients on original antihypertensive monotherapy ≤1080 days in 27 783 patients with all drug records exceed 1080 days)

	Without time-dependent effects			
Antihypertensive monotherapies	Crude HR (95% CI)	*P* value	IPW adjusted^a^ HR (95% CI)	*P* value
Reference: BBs				
Exposure				
ACEIs	0.83 (0.79;0.87)	<0.001	0.87 (0.82;0.93)	<0.001
ARBs	0.79 (0.73;0.85)	<0.001	0.82 (0.76;0.89)	<0.001
CCBs	1.05 (0.98;1.12)	0.166	1.11 (1.02;1.21)	0.017
Thiazides	0.85 (0.81;0.90)	<0.001	0.93 (0.88;0.98)	0.012
AUC at 1080 days	0.796		0.784	
Sensitivity	0.555		0.555	
Specificity	1		1	
Nagelkerke's *R*^2^	0.007		0.039	
Harrell's *C*-index	0.551		0.552	

	With time-dependent effects			
Exposure	Crude HR (95% CI)	*P* value	IPW adjusted^a^ HR (95% CI)	*P* value
ACEIs	1.12 (1.02;1.23)	0.021	1.01 (0.90;1.13)	0.911
ARBs	1.46 (1.21;1.76)	<0.001	1.11 (0.89;1.38)	0.355
CCBs	2.65 (2.04;3.45)	<0.001	1.73 (1.26;2.37)	<0.001
Thiazides	13.50 (6.35;28.73)	<0.001	3.51 (1.43;8.65)	0.006
tt (antihypertensive drug monotherapy)	0.68 (0.61;0.76)	<0.001	0.83 (0.73;0.94)	0.004
AUC at 1080 days	0.553		0.598	
Sensitivity	0.463		0.483	
Specificity	0.791		0.821	
Nagelkerke's *R*^2^	0.011		0.043	
Harrell's *C*-index	0.547		0.549	

a IPW adjusted between antihypertensive monotherapies and gender, age, drugs for diabetes, RA, asthma/COPD, antiepileptics, antiparkinson, psycholeptics, psychoanaleptic, addictive_disorders, antineoplastic, calendar-year periods. ACEIs, angiotensin converting enzyme inhibitors; ARBs, angiotensin II receptor blockers; BBs, beta-blockers; CCBs, calcium channel blockers; CI, confidence interval; HR, hazard ratio; IPW, inverse probability weighting.

### Sensitivity analysis

Logistic regression did not filter the variables, except for nonoutcome variables containing only one level of data. The main results remained the same as in LASSO-adjusted regression. The Hosmer–Lemeshow test showed that most models had a *P*-value >0.05, indicating a good model fit, although most Nagelkerke *R*^2^ values were low (see Tables 10–14, Supplemental Digital Content).

## DISCUSSION

The adherence rate from the first year to the tenth year had been increasing year by year. CCB users were more likely to continue the original treatment, and thiazide users were more likely to change the monotherapy.

ACEIs, ARBs, and thiazides, middle and older age, with initial diabetes, asthma/COPD drug, and without psycholeptics drug, calendar year after 2000 were contributing factors to high adherence. Thiazides and being male were harmful to continuation and helpful to discontinuation, switching and add on. Middle and older age promoted switch and add on but impeded discontinuation. High adherence contributed to continuation, switch and add on but impeded discontinuation.

Thiazide users showed an average lower likelihood of achieving high adherence in 3-year and higher likelihood at baseline compared to BB users, while CCB users showed a higher likelihood in both situation.

### High adherence rate in the Netherlands

Although the adherence to antihypertensive monotherapy is influenced by various factors [28], nonadherence remains a significant problem. The high adherence rate in this study was consistent with a previous study in the Netherlands [6]. Average adherence was higher for ACEIs and ARBs than for BBs, CCBs, and thiazides in the 1^st^ year, it might due to the differences in indications, side effects, and dosing schedules. The differences were not obvious in the 10^th^ year, because patients who had initial monotherapy greater than 10 year have tolerated and adapted to the medication.

The global adherence rate varies from 49.8% to over 90% [29–34]. Compared with these studies, the adherence rate in our study remained high, which might be related to several reasons. First, the guidelines for hypertension treatment and CVD management all suggest starting with monotherapy. Second, we restricted the minimum follow-up time from 1-year to 10-year, favouring high adherence. Third, the healthcare providers might prescribed repeated dispensing for these drugs. Fourth, mandatory health insurance in the Netherlands covers most costs for prescribed antihypertensive drugs [35]. In general, the failure to prescribe adequate antihypertensive therapy may play a more significant role in poor blood pressure control than patient nonadherence, and the majority of adults with hypertension are not controlled to goal with monotherapy.

### Drug patterns

More than half of the number of patients who initially received monotherapy found it easy to discontinue and <30% of patients remained on the initial monotherapy after a follow-up period of 1-year. This was consistent with the drug utilization trend [31] indicating that single pill combinations or combination therapy can improve adherence compared to monotherapy. The low continuation rate and high discontinuation rate of thiazides and BBs might suggest that they were used more often in short term treatment. While the opposite performance of CCBs suggested they might be better used in long term.

### High adherence risk prediction model

In our study, ACEIs, ARBs, and thiazides exhibited higher adherence rates compared to BBs. The results partially aligned with two studies [36,37], indicating that ACEIs and ARBs exhibited superior adherence compared to other medication classes. This suggests that compared to BBs, ACEIs, ARBs, and thiazides may be more tolerable for patients to improve adherence. From a clinical perspective, this finding can help healthcare providers choose medications that optimize adherence and improve the management of hypertension.

Older age was also associated with high chance of high adherence, consistent with research in China [37]. This was contrary to a study in Italy [38] which found that patients aged 85 years and over were most at risk of nonadherence. Older patients might have more time and patience to focus on their health condition and adhere to prescriptions. Additionally, the use of psycholeptics drug was not good to maintain high adherence, which was also consist with the common conclusion [39]. So, general practitioners and the clinicians should also provide additional support, such as medication counseling to consolidate hypertension management in the elders in case the cognitive decline.

Unlike research in Canada [34] and Italy [40], sex did not show a relation with high-adherence in our study, which indicates the need for personalized approaches to optimize patient care based on multiple factors, rather than sex alone.

The first version of cardiovascular risk management guidelines was published in 2006 [41]. Furthermore, it has been mandatory for Dutch citizens to possess health insurance provided by one of the Dutch health insurers since 2006 [35]. This change might improve adherence by lowering barriers.

Since most of the results in different clusters were the same as in all the patients, so the above discrepancy might not influenced by the sex and the year of the first exposure prescription.

### Drug pattern risk predict model

A study from China [42] concluded that BB users were more likely to discontinue compared to thiazide users, which was opposite to our findings, while CCB users were more likely to continue monotherapy. Since high adherence was related with both discontinuation and continuation, enhanced adherence can increase the drug retention rate. Furthermore, patients may be more likely to discontinue medications if they experience overlapping side effects or interactions between antihypertensive drugs and other comorbidities drugs. An interesting finding was that older age patients benefited the continuation rate after 2010, which may related to the insurance protection in the Netherlands.

A Swedish study [43] found that males were at a high risk of poor continuation with the initial drug, which was consistent with our results. Patients possessed high adherence to initial monotherapy, with middle or older age, or with diabetes drugs were easier to switch and add on other drugs, suggesting that they remain focused on their health and timely switched to or added on another drug if the results were not satisfactory. Thiazides was a risk factor for switch and add on regardless of sex and calendar year. The reason for changing treatments might simply be that people found the side effects of thiazide diuretics intolerable, as was seen in a Chinese study [44]. It was also consistent with the recommended combination therapy of ACEI or ARB with thiazides [45].

### Relative risk of high adherence

The results suggested that the time effect will influence the hazards though the effect decreasing over time. Thiazides were better for the short term use to achieve high adherence while CCBs in both short term and long term were better to achieve high adherence than BBs. This conclusion was also consist with the continuation rate of thiazides and CCBs.

## SUMMARY

In general, our findings would advance the personalized medicine can be applicable in the clinical setting, focusing on population with similar characteristics (elder, use of comorbid medications, etc.) and developing corresponding drug prevention strategy (suitable antihypertensive monotherapy) can improve drug adherence and other health outcome, such as reducing cardiovascular risk, saving financial budget.

### Strength and limitations

A comprehensive examination of antihypertensive drug monotherapy adherence and drug patterns is a key strength. Employing a clustering method, we categorized patients into distinct groups and the consistency of our main results within these clusters adds robustness to our conclusions. By ensuring that the results hold across diverse patient groups, we enhanced the reliability of our findings. The clusters remained the same after adjusting adherence, which eliminated the bias caused by adherence as a risk factor when entered into the drug pattern risk prediction model.

The utilization of LASSO logistic regression is another strength, because it allows for the selection of key variables and the simplification of the risk prediction model. This approach not only refined the model but also facilitated a clearer understanding of the factors influencing adherence and drug patterns.

However, it is crucial to acknowledge certain limitations in our study. One notable limitation is the insufficiency of variables available for predicting adherence and drug patterns, such as lifestyle, socioeconomic status, etc. While we employed rigorous methodologies, the absence of certain variables may restrict the depth of our predictive models. Furthermore, adherence in our study is higher than most previous reports. Thus, findings may not be generalizable. Though we already excluded some patients who used specific antihypertensive drug classes but not for CVD prevention based on our earlier research, for instance, BBs also be prescribed as preventive medication for migraine patients, there are still potential nonrelated patients we cannot distinguish from the prescription. Moreover, there is variability in adherence and other outcomes among individual medications within the same class of antihypertensive monotherapy. This variability may affect the generalizability of the findings, as some medications within a class could demonstrate better adherence and distinct utilization patterns compared to others. Understanding these differences is crucial for accurately interpreting the study's implications and applying them effectively in real-world clinical settings.

In general, pharmacy-based studies have been widely used in adherence research, as they provide objective medication usage patterns without relying on self-reported adherence, which can be subject to recall bias. While our findings do not encompass every possible clinical factor, they offer a foundational understanding of patient behaviour, which can guide targeted interventions to improve adherence and optimize treatment strategies. Future research endeavours should consider incorporating a broader range of variables to further enhance the accuracy and comprehensiveness of predictions related to adherence and drug patterns in antihypertensive monotherapy; incorporating clinical records and patient-reported outcomes could further enhance the applicability of our findings; expanding on comorbidity assessment by integrating electronic health record data for a more comprehensive risk stratification.

## CONCLUSION

The results highlight key factors influencing medication adherence and treatment changes, emphasizing the need for personalized approaches to optimize patient care. Enhancing adherence and provide the monotherapy for short term and long term plan were beneficial to better personalized treatment.

## ACKNOWLEDGEMENTS

We thank the pharmacies that supplied data to the University Groningen IADB.nl database.

Ethics approval and consent to participate: This study is based on established database IADB.nl. Data are collected in accordance with the national and European guidelines on privacy requirements for handling human data. The authors have no ethical conflicts to disclose. Ethics approval is not needed and required for this study.

Clinical trial number: Not applicable.

Consent for publication: Not applicable.

Availability of data and materials: The study remains in progress and the data are not currently available for sharing.

Funding: Xuechun Li is funded by the China Scholarship Council (file no: 202106070028).

Authors’ contributions: X.C.L. conceived and M.D.T., S.M., E.H., C.C.M.S.V. and J.H.J. designed the study. J.H.J. constructed data. S.M. provided statistical support. X.C.L., M.D.T., S.M., and E.H. wrote the first draft. All the authors reviewed and approved the final article.

### Conflicts of interest

The authors report no conflicts of interest in this work.

## Supplementary Material

Supplemental Digital Content
