# Supplementary material for: LASSO logistic regression and cluster analysis in predicting adherence and drug patterns among new users of monotherapy for antihypertensive drugs
Source: J Hypertens. 2025 Jun 20;43(9):1519–28. doi: 10.1097/HJH.0000000000004084 (PMC12337933; doi:10.1097/HJH.0000000000004084)
Supplement: Supplemental Digital Content [file jhype-43-1519-s001.pdf]

Supplementary table 1. Model comparison among three penalized regression models (Ridge, LASSO, Elastic Net) and logistic regression model.

	**Ridge**	**LASSO**	**Elastic Net**	**Logistic**
**RMSE**	0.3366182	0.3366118	0.3366101	/
**Rsquared**	0.01602122	0.01605109	0.01602435	/
**Auc**	/	0.601	/	0.5*/0.599**

* AUC was computed through simple cross-validation on the 7:3 data split

** Mean AUC was computed through a 10-fold cross-validation

Abbreviations: LASSO, Least Absolute Shrinkage and Selection Operator; RMSE, Root mean square error; AUC, area under the curve

Supplementary table 2. Adherence description in patients on original antihypertensive monotherapy exceeding 360 days (1 year),720 days(2 year),1080 days(3 year),1440 days(4 year),1800 days(5 year),2160 days(6 year),2520 days(7 year),2880 days(8 year),3240 days(9year),3600 days(10 year).

	**Average adherence(SDs)**						**Higher adherence (≥0.8)**
		ACEIs	ARBs	BBs	CCBs	Thiazides	
1-year, n=25568	0.92 (0.14)	0.94 (0.11)	0.93 (0.13)	0.90 (0.17)	0.91 (0.14)	0.92 (0.13)	22101 (86.4%)
2-year, n=17834	0.92 (0.14)	0.93 (0.12)	0.93 (0.14)	0.91 (0.16)	0.92 (0.13)	0.93 (0.12)	15638 (87.7 %)
3-year, n=13155	0.92 (0.13)	0.93 (0.12)	0.92 (0.14)	0.92 (0.15)	0.92 (0.13)	0.93 (0.11)	11673 (88.7%)
4-year, n=9835	0.93 (0.13)	0.94 (0.11)	0.93 (0.13)	0.92 (0.15)	0.93 (0.14)	0.93 (0.11)	8813 (89.6%)
5-year, n=7517	0.93 (0.13)	0.94 (0.11)	0.94 (0.11)	0.92 (0.15)	0.92 (0.15)	0.94 (0.10)	6789 (90.3%)
6-year, n=5777	0.94 (0.12)	0.94 (0.10)	0.95 (0.09)	0.93 (0.13)	0.94 (0.12)	0.94 (0.10)	5293 (91.6%)
7-year, n=4411	0.94 (0.11)	0.94 (0.09)	0.95 (0.08)	0.94 (0.13)	0.94 (0.13)	0.94 (0.09)	4052 (91.9%)
8-year, n=3391	0.94 (0.11)	0.94 (0.11)	0.94 (0.10)	0.94 (0.12)	0.94 (0.12)	0.94 (0.10)	3110 (91.7%)
9-year, n=2655	0.94 (0.10)	0.94 (0.11)	0.95 (0.08)	0.94 (0.11)	0.95 (0.09)	0.95 (0.09)	2467 (92.9%)

10-year, n=2086	0.94 (0.10)	0.94 (0.10)	0.95 (0.08)	0.95 (0.11)	0.95 (0.10)	0.94 (0.09)	1937 (92.9%)

Abbreviations: SDs, Standard deviations; ACEIs, Angiotensin converting enzyme inhibitors; ARBs, Angiotensin II receptor blockers; BBs, Beta-blockers; CCBs, Calcium channel blockers.

Supplementary table 3. General drug patterns in patients on original antihypertensive monotherapy exceeding 360 days (1 year). N (%).

	Overall (N=25568)	ACEIs (N=6260)	ARBs (N=2296)	BBs (N=9113)	CCBs (N=2322)	Thiazides (N=5577)
Continuation	6994 (27.4%)	1913 (30.6%)	684 (29.8%)	2335 (25.6%)	898 (38.7%)	1164 (20.9%)
Discontinuation	14515 (56.8%)	3385 (54.1%)	1251 (54.5%)	5264 (57.8%)	1161 (50.0%)	3454 (61.9%)
Switch	3963 (15.5%)	1050 (16.8%)	453 (19.7%)	1049 (11.5%)	247 (10.6%)	1164 (20.9%)
Add_on	8977 (35.1%)	2249 (35.9%)	847 (36.9%)	2944 (32.3%)	572 (24.6%)	2365 (42.4%)

Abbreviations: ACEIs, Angiotensin converting enzyme inhibitors; ARBs, Angiotensin II receptor blockers; BBs, Beta-blockers; CCBs, Calcium channel blockers.

Supplementary table 4. Clustering groups results

**Clustering groups**	**Characteristics**
**Outcome=adherence**	
**Cluster 1 (N=5091)**	Male;<br>Most patients use diabetes drugs;<br>Patients who use RA drugs the least;<br>Patients who use antiepileptics drugs the least;<br>Patients who use antiparkinson drugs the least;<br>Patients who use psychoanaleptics drugs the least;<br>Calendar-year before 2010
**Cluster 2 (N=7305)**	Female;<br>Patients who use asthma/COPD drugs the least;<br>Most patients use psycholeptics drugs;<br>Patients who use addictive_disorders drugs the least;<br>Patients who use antineoplastic drugs the least;<br>Calendar-year before 2010.
**Cluster 3 (N=6334)**	Male;<br>Patients who use psycholeptics drugs the least;<br>Most patients use addictive_disorders drugs;<br>Most patients use antineoplastic drugs drugs;<br>Calendar-year after 2010.
**Cluster 4 (N=6838)**	Female;<br>Patients who use diabetes drugs the least;<br>Most patients use RA drugs;<br>Most patients use asthma/COPD drugs;<br>Most patients use antiepileptics drugs;<br>Most patients use antiparkinson drugs;

	Most patients use psychoanaleptics drugs;<br>Calendar-year after 2010.
**Outcome= drug patterns**	Same clusters assignment as when outcome=adherence.

Abbreviations: RA, Rheumatoid arthritis; COPD, Chronic obstructive pulmonary disease

Supplementary table 5. LASSO logistic regression analysis of risk factors for high adherence in patients on original antihypertensive monotherapy exceeding 360 days (1 year)

	High adherence		Cluster 1		Cluster 2		Cluster 3		Cluster 4	
**Anti-hypertensive drug class**	OR(95% CI)	p								
BBs(reference)	1		1		1		1		1	
ACEIs	1.66(1.49~1.84)	<0.001	1.32(1.07~1.64)	0.012	1.55(1.25~1.94)	<0.001	2.05(1.68~2.5)	<0.001	2.05(1.66~2.54)	<0.001
ARBs	1.53(1.33~1.78)	<0.001	1.19(0.9~1.6)	0.226	1.2(0.92~1.59)	0.189	1.9(1.43~2.57)	<0.001	2.36(1.72~3.32)	<0.001
CCBs	1.03(0.91~1.18)	0.641	0.73(0.52~1.03)	0.070	0.86(0.64~1.16)	0.305	1.32(1.04~1.68)	0.022	1.31(1.04~1.65)	0.022
Thiazides	1.23(1.12~1.36)	<0.001	1.07(0.86~1.35)	0.547	0.96(0.82~1.12)	0.578	1.71(1.35~2.16)	<0.001	1.72(1.4~2.12)	<0.001
**Sex**										
Female(reference)	1		-	-	-	-	-	-	-	-
Male	0.95(0.89~1.03)	0.219	-	-	-	-	-	-	-	-
**Age(year)**										
18-39(reference)	1		1		1		1		1	
40-69	1.93(1.74~2.13)	<0.001	1.52(1.19~1.92)	<0.001	1.97(1.64~2.36)	<0.001	1.87(1.5~2.33)	<0.001	2.15(1.76~2.61)	<0.001
≥70	2.26(1.99~2.57)	<0.001	1.5(1.11~2.01)	0.007	2.09(1.67~2.6)	<0.001	2.69(2.02~3.6)	<0.001	3.14(2.42~4.1)	<0.001
**Diabetes drug**										
No(reference)	1		1		1		1		1	
Yes	1.21(1.01~1.46)	0.039	0.99(0.75~1.35)	0.973	1.41(1.02~1.99)	0.041	1.75(1.14~2.84)	0.016	1.07(0.67~1.8)	0.795
**RA drug**										
No(reference)	1		-	-	1		1		1	
Yes	1.32(0.87~2.09)	0.213	-	-	0.66(0.35~1.36)	0.222	8.23(1.81~145.69)	0.037	1.14(0.58~2.59)	0.727

**Asthma/COPD drug**										
No(reference)	1		1		-	-	1		1	
Yes	1.21(1.04~1.4)	0.012	1.39(1~1.99)	0.059	-	-	1.16(0.87~1.58)	0.316	1.43(1.08~1.93)	0.016
**Antiepileptics**										
No(reference)	1		-	-	-	-	1		1	
Yes	1.21(0.94~1.59)	0.156	-	-	-	-	1.36(0.8~2.5)	0.286	1.39(0.86~2.38)	0.209
**Antiparkinson**										
No(reference)	1		1		1		-	-	1	
Yes	1.24(0.78~2.09)	0.382	2.15(0.62~13.52)	0.304	1.58(0.68~4.62)	0.341	-	-	1.18(0.53~3.15)	0.707
**Psycholeptics**										
No(reference)	1		1		1		1		1	
Yes	0.78(0.71~0.85)	<0.001	0.65(0.53~0.81)	<0.001	0.78(0.67~0.91)	0.001	1.19(0.92~1.56)	0.196	0.71(0.59~0.85)	<0.001
**Psychoanaleptic**										
No(reference)	-	-	-	-	-	-	-	-	-	-
Yes	-	-	-	-	-	-	-	-	-	-
**Addictive_disorders**										
No(reference)	1		1		1		-	-	1	
Yes	1.31(0.8~2.31)	0.316	1.9(0.54~12.01)	0.394	0.95(0.3~4.21)	0.938	-	-	1.56(0.62~5.25)	0.401
**Antineoplastic**										

No(reference)	1		-	-	1		-	-	-	-
Yes	0.83(0.45~1.67)	0.564	-	-	0.6(0.21~2.17)	0.385	-	-	-	-
**Calendar year**										
1996-2000(reference)	1		-	-	1		-	-	-	-
2000-2010	1.19(1.03~1.36)	0.015	-	-	1.21(1.02~1.44)	0.028	-	-	-	-
2010-2020	1.35(1.18~1.55)	<0.001	-	-	-	-	-	-	-	-
**AUC**	0.601		0.560		0.575		0.613		0.648	
**Sensitivity(true positive rate)**	1		1		1		1		1	
**Specificity(true negative rate)**	0		0		0		0		0	
**Hosmer-Lemeshow test**	0.221		0.464		0.526		0.882		0.023	
**Nagelkerke R^2**	0.032		0.017		0.026		0.042		0.053	

Reference group/category is set to 1.

-: No relevant data available.

Abbreviations: LASSO, Least Absolute Shrinkage and Selection Operator; OR, Odds ratio; CI, Confidence interval; ACEIs, Angiotensin converting enzyme inhibitors; ARBs, Angiotensin II receptor blockers; BBs, Beta-blockers; CCBs, Calcium channel blockers; RA, Rheumatoid arthritis; COPD, Chronic obstructive pulmonary disease; AUC, area under the curve

Supplementary table 6. LASSO logistic regression analysis of risk factors for continuation in patients on original antihypertensive monotherapy exceeding 360 days (1 year)

	Continuation		Cluster 1		Cluster 2		Cluster 3		Cluster 4	
**Anti-hypertensive drug class**	OR(95% CI)	P	OR(95% CI)	P	OR(95% CI)	p	OR(95% CI)	p	OR(95% CI)	p
BBs(reference)	1		1		1		1		1	
ACEIs	1.01(0.94~1.09)	0.782	1.11(0.91~1.36)	0.302	0.97(0.8~1.17)	0.725	1.06(0.92~1.22)	0.393	1(0.87~1.14)	0.958
ARBs	1.01(0.91~1.12)	0.872	1.1(0.84~1.44)	0.473	0.93(0.72~1.18)	0.539	1.02(0.84~1.24)	0.812	1.04(0.86~1.25)	0.715
CCBs	1.25(1.13~1.39)	<0.001	1.02(0.68~1.48)	0.921	0.83(0.59~1.13)	0.248	1.4(1.17~1.66)	<0.001	1.35(1.15~1.59)	<0.001
Thiazides	0.65(0.6~0.71)	<0.001	0.63(0.49~0.81)	<0.001	0.63(0.53~0.75)	<0.001	0.63(0.53~0.74)	<0.001	0.7(0.61~0.81)	<0.001
**Adherence**										
Low(reference)	1		1		1		1		1	
High	1.82(1.66~2)	<0.001	1.81(1.4~2.39)	<0.001	1.68(1.38~2.07)	<0.001	2.06(1.74~2.45)	<0.001	1.64(1.4~1.93)	<0.001
**Sex**										
Female(reference)	1		-	-	-	-	-	-	-	-
Male	0.94(0.88~0.99)	0.029	-	-	-	-	-	-	-	-
**Age**										
18-39(reference)	1		1		1		1		1	
40-69	1.4(1.26~1.55)	<0.001	0.85(0.67~1.1)	0.216	1.42(1.15~1.77)	0.001	1.41(1.17~1.7)	<0.001	1.66(1.39~1.97)	<0.001
≥70	1.1(0.98~1.24)	0.117	0.46(0.33~0.64)	<0.001	0.67(0.52~0.87)	0.003	1.36(1.1~1.69)	0.005	1.62(1.33~1.98)	<0.001
**Diabetes drug**										
No(reference)	1		1		1		1		1	
Yes	0.78(0.68~0.9)	<0.001	0.76(0.56~1.02)	0.074	0.72(0.53~0.97)	0.034	0.98(0.78~1.24)	0.890	0.62(0.45~0.84)	0.003
**RA drug**										

No(reference)	1		-	-	1		1		-	-
Yes	0.88(0.65~1.17)	0.382	-	-	0.53(0.18~1.23)	0.181	0.98(0.59~1.6)	0.928	-	-
**Asthma/COPD drug**										
No(reference)	1		-	-	1		-	-	-	-
Yes	0.94(0.85~1.05)	0.271	-	-	1.08(0.83~1.38)	0.553	-	-	-	-
**Antiepileptics**										
No(reference)	1		1		1		-	-	1	
Yes	0.94(0.77~1.15)	0.567	0.49(0.21~1)	0.077	1.2(0.76~1.84)	0.416	-	-	1(0.72~1.37)	0.984
**Antiparkinson**										
No(reference)	1		-	-	-	-	1		1	
Yes	0.7(0.47~1.01)	0.067	-	-	-	-	0.57(0.27~1.12)	0.117	0.63(0.33~1.14)	0.140
**Psycholeptics**										
No(reference)	1		-	-	1		1		1	
Yes	0.86(0.79~0.94)	<0.001	-	-	0.93(0.79~1.09)	0.359	0.82(0.68~0.98)	0.030	0.8(0.7~0.92)	0.002
**Psychoanaleptic**										
No(reference)	-	-	-	-	-	-	-	-	-	-
Yes	-	-	-	-	-	-	-	-	-	-
**Addictive_disorders**										
No(reference)	1		-	-	-	-	1		-	-

Yes	0.77(0.53~1.12)	0.182	-	-	-	-	0.64(0.35~1.1)	0.113	-	-
**Antineoplastic**										
No(reference)	1		-	-	1		1		1	
Yes	0.76(0.42~1.3)	0.331	-	-	2.84(0.88~7.9)	0.056	0.56(0.2~1.39)	0.237	0.54(0.18~1.42)	0.244
**Calendar year**										
1996-2000(reference)	1		1		1		-	-	-	-
2000-2010	1.11(0.96~1.28)	0.172	1.17(0.91~1.51)	0.231	1.06(0.89~1.27)	0.508	-	-	-	-
2010-2020	3.67(3.19~4.23)	<0.001	-	-	-	-	-	-	-	-
**AUC**	0.684		0.592		0.590		0.579		0.559	
**Sensitivity(true positive rate)**	0		0		0		0.112		0.001	
**Specificity(true negative rate)**	1		1		1		0.933		0.998	
**Hosmer-Lemeshow test**	0.285		0.059		0.018		0.758		0.009	
**Nagelkerke R^2**	0.124		0.024		0.044		0.039		0.036	

Reference group/category is set to 1.

-: No relevant data available.

Abbreviations: LASSO, Least Absolute Shrinkage and Selection Operator; OR, Odds ratio; CI, Confidence interval; ACEIs, Angiotensin converting enzyme inhibitors; ARBs, Angiotensin II receptor blockers; BBs, Beta-blockers; CCBs, Calcium channel blockers; RA, Rheumatoid arthritis; COPD, Chronic obstructive pulmonary disease; AUC, area under the curve

Supplementary table 7. LASSO logistic regression analysis of risk factors for discontinuation in patients on original antihypertensive monotherapy exceeding 360 days (1 year)

	Discontinuation		Cluster 1		Cluster 2		Cluster 3		Cluster 4	
**Anti-hypertensive drug class**	OR(95% CI)	p	OR(95% CI)	P	OR(95% CI)	p	OR(95% CI)	p	OR(95% CI)	p
BBs(reference)	1		1		1		1		1	
ACEIs	1.05(0.98~1.12)	0.186	1.08(0.92~1.26)	0.344	1.17(1.01~1.36)	0.041	0.85(0.74~0.97)	0.020	1.04(0.91~1.19)	0.593
ARBs	1.05(0.95~1.16)	0.321	1.18(0.96~1.46)	0.122	1.37(1.13~1.68)	0.002	0.83(0.69~1)	0.055	0.87(0.72~1.05)	0.153
CCBs	0.95(0.86~1.04)	0.266	1.22(0.91~1.65)	0.184	1.42(1.11~1.83)	0.006	0.72(0.6~0.86)	<0.001	0.82(0.7~0.97)	0.017
Thiazides	1.37(1.28~1.48)	<0.001	1.4(1.17~1.66)	<0.001	1.62(1.43~1.84)	<0.001	1.22(1.04~1.43)	0.016	1.17(1.03~1.34)	0.019
**Adherence**										
Low(reference)	1		1		1		1		1	
High	0.47(0.44~0.51)	<0.001	0.47(0.38~0.56)	<0.001	0.48(0.41~0.56)	<0.001	0.44(0.37~0.52)	<0.001	0.53(0.45~0.61)	<0.001
**Sex**										
Female(reference)	1		-	-	-	-	-	-	-	-
Male	1.06(1~1.11)	0.039	-	-	-	-	-	-	-	-
**Age**										
18-39(reference)	1		1		1		1		1	
40-69	0.5(0.45~0.54)	<0.001	0.65(0.52~0.79)	<0.001	0.44(0.37~0.53)	<0.001	0.54(0.45~0.65)	<0.001	0.46(0.39~0.54)	<0.001
≥70	0.72(0.65~0.8)	<0.001	1.2(0.93~1.54)	0.166	0.81(0.66~0.99)	0.043	0.67(0.54~0.82)	<0.001	0.51(0.42~0.61)	<0.001
**Diabetes drug**										
No(reference)	1		1		1		1		1	
Yes	1.08(0.96~1.21)	0.182	1.03(0.83~1.28)	0.797	1.11(0.89~1.38)	0.357	0.95(0.76~1.2)	0.685	1.24(0.93~1.67)	0.148
**RA drug**										

No(reference)	-	-	1		1		1		1	
Yes	-	-	1.2(0.58~2.67)	0.638	1.57(0.86~3.05)	0.159	0.97(0.58~1.58)	0.890	0.77(0.49~1.2)	0.256
**Asthma/COPD drug**										
No(reference)	1		1		-	-	1		-	-
Yes	1.11(1.01~1.23)	0.027	1.21(0.95~1.55)	0.123	-	-	1.1(0.92~1.32)	0.302	-	-
**Antiepileptics**										
No(reference)	1		1		1		1		1	
Yes	1.1(0.92~1.33)	0.284	1.85(1.13~3.19)	0.019	0.88(0.61~1.27)	0.484	1.12(0.79~1.6)	0.508	1(0.73~1.38)	0.976
**Antiparkinson**										
No(reference)	1		1		1		1		1	
Yes	1.75(1.25~2.5)	0.001	5.06(1.47~31.81)	0.029	1.58(0.81~3.33)	0.202	2.24(1.17~4.51)	0.018	1.2(0.69~2.11)	0.523
**Psycholeptics**										
No(reference)	1		1		1		1		1	
Yes	1.2(1.11~1.29)	<0.001	1.14(0.95~1.38)	0.157	1.06(0.94~1.2)	0.335	1.35(1.13~1.62)	0.001	1.32(1.15~1.52)	<0.001
**Psychoanaleptic**										
No(reference)	1		1		1		1		1	
Yes	1.09(1~1.19)	0.051	1.19(0.93~1.52)	0.176	1.26(1.07~1.48)	0.006	0.92(0.76~1.12)	0.422	1.01(0.87~1.17)	0.921
**Addictive_disorders**										
No(reference)	1		1		1		1		-	-

Yes	1.26(0.9~1.78)	0.184	1.86(0.72~5.74)	0.230	1.75(0.61~6.27)	0.335	1.52(0.9~2.58)	0.116	-	-
**Antineoplastic**										
No(reference)	1		1		1		1		1	
Yes	1.24(0.76~2.04)	0.392	1.5(0.44~6.84)	0.549	0.5(0.18~1.35)	0.160	1.91(0.81~4.83)	0.150	1.22(0.49~3.09)	0.671
**Calendar year**										
1996-2000(reference)	1		-	-	1		-	-	-	-
2000-2010	0.95(0.85~1.06)	0.329	-	-	0.93(0.81~1.06)	0.275	-	-	-	-
2010-2020	0.49(0.44~0.54)	<0.001	-	-	-	-	-	-	-	-
**AUC**	0.635		0.596		0.630		0.585		0.573	
**Sensitivity(true positive rate)**	0.736		1		0.996		0.332		0.333	
**Specificity(true negative rate)**	0.446		0		0.003		0.804		0.776	
**Hosmer-Lemeshow test**	0.483		0.079		<0.001		0.548		0.214	
**Nagelkerke R^2**	0.083		0.048		0.065		0.056		0.046	

Reference group/category is set to 1.

-: No relevant data available.

Abbreviations: LASSO, Least Absolute Shrinkage and Selection Operator; OR, Odds ratio; CI, Confidence interval; ACEIs, Angiotensin converting enzyme inhibitors; ARBs, Angiotensin II receptor blockers; BBs, Beta-blockers; CCBs, Calcium channel blockers; RA, Rheumatoid arthritis; COPD, Chronic obstructive pulmonary disease; AUC, area under the curve

Supplementary table 8. LASSO logistic regression analysis of risk factors for switch in patients on original antihypertensive monotherapy exceeding 360 days (1 year)

	Switch		Cluster 1		Cluster 2		Cluster 3		Cluster 4	
**Anti-hypertensive drug class**	OR(95% CI)	p	OR(95% CI)	P	OR(95% CI)	p	OR(95% CI)	p	OR(95% CI)	p
BBs(reference)	1		1		1		1		1	
ACEIs	1.65(1.49~1.82)	<0.001	1.36(1.14~1.63)	<0.001	1.88(1.57~2.24)	<0.001	1.8(1.41~2.3)	<0.001	1.81(1.45~2.28)	<0.001
ARBs	1.99(1.76~2.25)	<0.001	2.26(1.81~2.8)	<0.001	2.65(2.14~3.28)	<0.001	1.57(1.13~2.17)	0.007	1.24(0.88~1.72)	0.201
CCBs	1.17(1~1.36)	0.041	1.12(0.8~1.56)	0.492	1.38(1.01~1.86)	0.036	1.28(0.93~1.75)	0.129	1.06(0.78~1.42)	0.714
Thiazides	2.07(1.88~2.27)	<0.001	1.89(1.57~2.26)	<0.001	1.95(1.68~2.27)	<0.001	3.06(2.37~3.96)	<0.001	1.97(1.58~2.47)	<0.001
**Adherence**										
Low(reference)	1		1		1		-	-	-	-
High	1.28(1.15~1.43)	<0.001	1.36(1.12~1.67)	0.003	1.36(1.14~1.64)	<0.001	-	-	-	-
**Sex**										
Female(reference)	1		-	-	-	-	-	-	-	-
Male	1.23(1.14~1.32)	<0.001	-	-	-	-	-	-	-	-
**Age**										
18-39(reference)	1		1		1		1		1	
40-69	1.47(1.28~1.68)	<0.001	1.44(1.14~1.85)	0.003	1.68(1.33~2.14)	<0.001	1.23(0.9~1.71)	0.204	1.43(1.04~1.99)	0.031
≥70	1.57(1.35~1.83)	<0.001	1.22(0.93~1.63)	0.158	1.9(1.48~2.47)	<0.001	1.5(1.06~2.16)	0.024	1.71(1.21~2.45)	0.003
**Diabetes drug**										
No(reference)	1		1		1		-	-	-	-
Yes	1.15(1~1.31)	0.051	1.21(0.97~1.51)	0.095	1.27(1.01~1.59)	0.041	-	-	-	-
**RA drug**										

No(reference)	1		1		1		1		1	
Yes	0.81(0.54~1.19)	0.311	0.6(0.2~1.44)	0.292	0.88(0.4~1.75)	0.734	0.51(0.15~1.25)	0.196	1.36(0.68~2.49)	0.345
**Asthma/COPD drug**										
No(reference)	1		-	-	1		-	-	1	
Yes	1.07(0.94~1.22)	0.278	-	-	1.02(0.81~1.28)	0.847	-	-	1.37(1.06~1.76)	0.015
**Antiepileptics**										
No(reference)	-	-	-	-	1		-	-	1	
Yes	-	-	-	-	0.92(0.56~1.44)	0.713	-	-	0.62(0.3~1.12)	0.145
**Antiparkinson**										
No(reference)	-	-	-	-	1		-	-	-	-
Yes	-	-	-	-	0.39(0.12~0.98)	0.077	-	-	-	-
**Psycholeptics**										
No(reference)	1		-	-	1		-	-	-	-
Yes	1.03(0.93~1.13)	0.592	-	-	0.97(0.83~1.12)	0.680	-	-	-	-
**Psychoanaleptic**										
No(reference)	1		-	-	1		1		1	
Yes	0.88(0.78~1)	0.054	-	-	0.97(0.79~1.18)	0.737	0.76(0.54~1.05)	0.105	0.71(0.53~0.93)	0.017
**Addictive_disorders**										
No(reference)	-	-	1		1		-	-	-	-

Yes	-	-	0.56(0.13~1.67)	0.354	1.9(0.59~5.31)	0.242	-	-	-	-
**Antineoplastic**										
No(reference)	1		1		1		1		-	-
Yes	0.73(0.32~1.46)	0.416	0.3(0.02~1.54)	0.248	0.26(0.01~1.29)	0.194	1.9(0.54~5.21)	0.253	-	-
**Calendar year**										
1996-2000(reference)	1		-	-	1		-	-	-	-
2000-2010	0.89(0.79~1.01)	0.078	-	-	0.82(0.7~0.96)	0.014	-	-	-	-
2010-2020	0.37(0.33~0.42)	<0.001	-	-	-	-	-	-	-	-
**AUC**	0.655		0.580		0.616		0.608		0.598	
**Sensitivity(true positive rate)**	0		0		0		0		0	
**Specificity(true negative rate)**	1		1		0.999		1		1	
**Hosmer-Lemeshow test**	0.225		0.307		0.013		0.319		0.086	
**Nagelkerke R^2**	0.069		0.035		0.050		0.026		0.025	

Reference group/category is set to 1.

-: No relevant data available.

Abbreviations: LASSO, Least Absolute Shrinkage and Selection Operator; OR, Odds ratio; CI, Confidence interval; ACEIs, Angiotensin converting enzyme inhibitors; ARBs, Angiotensin II receptor blockers; BBs, Beta-blockers; CCBs, Calcium channel blockers; RA, Rheumatoid arthritis; COPD, Chronic obstructive pulmonary disease; AUC, area under the curve

Supplementary table 9. LASSO logistic regression analysis of risk factors for add on in patients on original antihypertensive monotherapy exceeding 360 days (1 year)

	Add on		Cluster 1		Cluster 2		Cluster 3		Cluster 4	
**Anti-hypertensive drug class**	OR(95% CI)	p	OR(95% CI)	P	OR(95% CI)	p	OR(95% CI)	p	OR(95% CI)	p
BBs(reference)	1		1		1		1		1	
ACEIs	1.2(1.11~1.29)	<0.001	1.07(0.93~1.24)	0.354	1.12(0.97~1.29)	0.135	1.56(1.32~1.83)	<0.001	1.3(1.1~1.52)	0.001
ARBs	1.22(1.1~1.35)	<0.001	1.16(0.95~1.42)	0.146	1.21(1~1.46)	0.048	1.46(1.17~1.81)	<0.001	1.3(1.05~1.61)	0.015
CCBs	0.84(0.75~0.94)	0.002	0.85(0.65~1.11)	0.243	0.92(0.73~1.17)	0.503	1.04(0.84~1.28)	0.732	0.84(0.68~1.02)	0.086
Thiazides	1.49(1.38~1.6)	<0.001	1.28(1.09~1.51)	0.002	1.13(1.01~1.27)	0.040	2.49(2.08~2.98)	<0.001	1.82(1.57~2.12)	<0.001
**Adherence**										
Low(reference)	1		1		1		1		1	
High	1.71(1.57~1.87)	<0.001	1.84(1.56~2.17)	<0.001	1.89(1.65~2.18)	<0.001	1.44(1.19~1.74)	<0.001	1.48(1.22~1.8)	<0.001
**Sex**										
Female(reference)	1		-	-	-	-	-	-	-	-
Male	1.14(1.08~1.2)	<0.001	-	-	-	-	-	-	-	-
**Age**										
18-39(reference)	1		1		1		1		1	
40-69	2.41(2.17~2.68)	<0.001	2.48(2.03~3.06)	<0.001	2.68(2.25~3.21)	<0.001	1.98(1.57~2.54)	<0.001	2.22(1.76~2.83)	<0.001
≥70	2.46(2.19~2.77)	<0.001	2.13(1.68~2.71)	<0.001	3.22(2.65~3.92)	<0.001	1.94(1.49~2.55)	<0.001	2.25(1.74~2.93)	<0.001
**Diabetes drug**										
No(reference)	1		1		1		-	-	1	
Yes	1.33(1.19~1.49)	<0.001	1.54(1.26~1.88)	<0.001	1.38(1.13~1.69)	0.002	-	-	1.42(1.03~1.93)	0.028
**RA drug**										

No(reference)	1		1		-	-	-	-	1	
Yes	1.14(0.86~1.49)	0.366	1.08(0.53~2.19)	0.833	-	-	-	-	1.68(1.05~2.64)	0.027
**Asthma/COPD drug**										
No(reference)	1		-	-	1		-	-	1	
Yes	1.04(0.94~1.15)	0.456	-	-	0.97(0.8~1.18)	0.778	-	-	1.13(0.93~1.37)	0.201
**Antiepileptics**										
No(reference)	1		1		-	-	1		1	
Yes	0.89(0.73~1.08)	0.248	0.89(0.58~1.36)	0.589	-	-	0.81(0.52~1.22)	0.324	0.78(0.52~1.14)	0.206
**Antiparkinson**										
No(reference)	1		1		1		1		1	
Yes	0.56(0.38~0.81)	0.003	0.46(0.18~1.08)	0.087	0.34(0.16~0.66)	0.003	0.4(0.14~0.95)	0.061	1.28(0.67~2.32)	0.437
**Psycholeptics**										
No(reference)	1		1		-	-	1		1	
Yes	0.99(0.92~1.07)	0.793	0.93(0.78~1.1)	0.375	-	-	0.87(0.7~1.06)	0.175	1.03(0.87~1.21)	0.722
**Psychoanaleptic**										
No(reference)	1		1		1		-	-	1	
Yes	0.86(0.78~0.94)	0.002	0.81(0.65~1.02)	0.077	0.91(0.78~1.06)	0.207	-	-	0.76(0.63~0.92)	0.004
**Addictive_disorders**										
No(reference)	1		-	-	-	-	-	-	-	-

Yes	1.03(0.71~1.48)	0.877	-	-	-	-	-	-	-	-
**Antineoplastic**										
No(reference)	1		1		1		-	-	1	
Yes	0.85(0.5~1.41)	0.537	0.66(0.2~2)	0.467	0.7(0.24~1.87)	0.484	-	-	1.36(0.47~3.5)	0.545
**Calendar year**										
1996-2000(reference)	1	-	1		-	-	-	-	-	-
2000-2010	1.01(0.91~1.12)	0.871	0.99(0.83~1.17)	0.880	-	-	-	-	-	-
2010-2020	0.4(0.36~0.45)	<0.001	-	-	-	-	-	-	-	-
**AUC**	0.667		0.577		0.593		0.611		0.602	
**Sensitivity(true positive rate)**	0.238		0.656		0.250		0		0	
**Specificity(true negative rate)**	0.885		0.450		0.814		1		1	
**Hosmer-Lemeshow test**	<0.001		0.128		0.080		0.769		0.199	
**Nagelkerke R^2**	0.100		0.058		0.058		0.047		0.048	

Reference group/category is set to 1.

-: No relevant data available.

Abbreviations: LASSO, Least Absolute Shrinkage and Selection Operator; OR, Odds ratio; CI, Confidence interval; ACEIs, Angiotensin converting enzyme inhibitors; ARBs, Angiotensin II receptor blockers; BBs, Beta-blockers; CCBs, Calcium channel blockers; RA, Rheumatoid arthritis; COPD, Chronic obstructive pulmonary disease; AUC, area under the curve

Supplementary table 10. Logistic regression analysis of risk factors for high adherence in patients on original antihypertensive monotherapy exceeding 360 days (1 year)

	High adherence		Cluster 1		Cluster 2		Cluster 3		Cluster 4	
**Anti-hypertensive drug class**	OR(95% CI)	p								
BBs(reference)	1		1		1		1		1	
ACEIs	1.66(1.49~1.84)	<0.001	1.31(1.06~1.63)	0.013	1.55(1.25~1.94)	<0.001	2.05(1.68~2.5)	<0.001	2.05(1.66~2.55)	<0.001
ARBs	1.53(1.33~1.78)	<0.001	1.19(0.9~1.6)	0.241	1.2(0.92~1.59)	0.190	1.9(1.43~2.56)	<0.001	2.36(1.72~3.32)	<0.001
CCBs	1.03(0.91~1.18)	0.640	0.73(0.52~1.03)	0.067	0.86(0.64~1.16)	0.305	1.32(1.04~1.68)	0.023	1.31(1.04~1.65)	0.021
Thiazides	1.24(1.12~1.36)	<0.001	1.06(0.85~1.34)	0.600	0.96(0.82~1.12)	0.582	1.71(1.35~2.16)	<0.001	1.72(1.4~2.13)	<0.001
**Sex**										
Female(reference)	1		-	-	-	-	-	-	-	-
Male	0.95(0.89~1.03)	0.223	-	-	-	-	-	-	-	-
**Age(year)**										
18-39(reference)	1		1		1		1		1	
40-69	1.93(1.74~2.13)	<0.001	1.51(1.19~1.92)	<0.001	1.97(1.63~2.36)	<0.001	1.88(1.5~2.33)	<0.001	2.15(1.77~2.61)	<0.001
≥70	2.26(1.99~2.57)	<0.001	1.5(1.11~2.02)	0.007	2.09(1.67~2.6)	<0.001	2.71(2.03~3.62)	<0.001	3.15(2.42~4.11)	<0.001
**Diabetes drug**										
No(reference)	1		1		1		1		1	
Yes	1.21(1.01~1.46)	0.039	1(0.75~1.35)	0.976	1.41(1.03~1.99)	0.041	1.75(1.14~2.84)	0.016	1.07(0.67~1.8)	0.794
**RA drug**										
No(reference)	1		1		1		1		1	
Yes	1.32(0.87~2.09)	0.213	1.6(0.56~6.73)	0.439	0.66(0.35~1.36)	0.226	8.24(1.81~145.78)	0.037	1.15(0.58~2.61)	0.715

**Asthma/COPD drug**										
No(reference)	1		1		1		1		1	
Yes	1.21(1.04~1.4)	0.012	1.4(1~2)	0.056	1.01(0.78~1.33)	0.923	1.17(0.87~1.59)	0.314	1.43(1.08~1.93)	0.016
**Antiepileptics**										
No(reference)	1		1		1		1		1	
Yes	1.21(0.94~1.59)	0.159	1.34(0.73~2.69)	0.373	0.95(0.61~1.54)	0.822	1.36(0.8~2.5)	0.292	1.38(0.85~2.37)	0.211
**Antiparkinson**										
No(reference)	1		1		1		1		1	
Yes	1.24(0.78~2.09)	0.383	2.13(0.62~13.39)	0.310	1.6(0.68~4.68)	0.332	0.84(0.37~2.26)	0.700	1.18(0.53~3.16)	0.705
**Psycholeptics**										
No(reference)	1		1		1		1		1	
Yes	0.77(0.7~0.85)	<0.001	0.66(0.53~0.82)	<0.001	0.78(0.67~0.91)	0.002	1.19(0.91~1.57)	0.219	0.7(0.58~0.85)	<0.001
**Psychoanaleptic**										
No(reference)	1		1		1		1		1	
Yes	1.01(0.9~1.14)	0.849	0.96(0.72~1.31)	0.795	1.02(0.83~1.26)	0.869	1.02(0.77~1.36)	0.902	1.04(0.84~1.29)	0.747
**Addictive_disorders**										
No(reference)	1		1		1		1		1	
Yes	1.31(0.8~2.3)	0.318	1.93(0.55~12.23)	0.381	0.95(0.3~4.19)	0.934	1.15(0.55~2.81)	0.733	1.55(0.62~5.22)	0.408
**Antineoplastic**										

No(reference)	1		1		1		1		1	
Yes	0.83(0.45~1.67)	0.566	2.04(0.39~37.45)	0.496	0.61(0.21~2.18)	0.390	0.89(0.3~3.86)	0.860	0.83(0.27~3.61)	0.769
**Calendar year**										
1996-2000(reference)	1		1		1		-	-	-	-
2000-2010	1.18(1.03~1.36)	0.016	1.17(0.92~1.46)	0.188	1.21(1.02~1.44)	0.029	-	-	-	-
2010-2020	1.35(1.18~1.55)	<0.001	-	-	-	-	-	-	-	-
**AUC**	0.5		0.5		0.5		0.5		0.5	
**Sensitivity(true positive rate)**	-		-		-		-		-	
**Specificity(true negative rate)**	-		-		-		-		-	
**Hosmer-Lemeshow test**	0.267		0.357		0.366		0.941		0.027	
**Nagelkerke R^2**	0.032		0.019		0.027		0.043		0.054	

Reference group/category is set to 1.

-: No relevant data available.

Abbreviations: OR, Odds ratio; CI, Confidence interval; ACEIs, Angiotensin converting enzyme inhibitors; ARBs, Angiotensin II receptor blockers; BBs, Beta-blockers; CCBs, Calcium channel blockers; RA, Rheumatoid arthritis; COPD, Chronic obstructive pulmonary disease; AUC, area under the curve

Supplementary table 11. Logistic analysis of risk factors for continuation in patients on original antihypertensive monotherapy exceeding 360 days (1 year)

	Continuation		Cluster 1		Cluster 2		Cluster 3		Cluster 4	
**Anti-hypertensive drug class**	OR(95% CI)	P	OR(95% CI)	P	OR(95% CI)	p	OR(95% CI)	p	OR(95% CI)	p
BBs(reference)	1		1		1		1		1	
ACEIs	1.01(0.94~1.09)	0.782	1.12(0.92~1.37)	0.253	0.97(0.8~1.17)	0.715	1.06(0.92~1.22)	0.406	1(0.87~1.15)	0.993
ARBs	1.01(0.91~1.12)	0.872	1.11(0.85~1.45)	0.431	0.92(0.72~1.18)	0.524	1.02(0.85~1.24)	0.809	1.04(0.86~1.26)	0.676
CCBs	1.25(1.13~1.39)	<0.001	1.05(0.7~1.53)	0.801	0.83(0.59~1.13)	0.245	1.4(1.18~1.66)	<0.001	1.36(1.16~1.59)	<0.001
Thiazides	0.65(0.6~0.71)	<0.001	0.64(0.49~0.82)	<0.001	0.63(0.53~0.74)	<0.001	0.63(0.53~0.74)	<0.001	0.71(0.61~0.81)	<0.001
**Adherence**										
Low(reference)	1		1		1		1		1	
High	1.82(1.66~2)	<0.001	1.83(1.41~2.41)	<0.001	1.68(1.38~2.07)	<0.001	2.06(1.74~2.46)	<0.001	1.64(1.4~1.93)	<0.001
**Sex**										
Female(reference)	1		-	-	-	-	-	-	-	-
Male	0.94(0.88~0.99)	0.029	-	-	-	-	-	-	-	-
**Age**										
18-39(reference)	1		1		1		1		1	
40-69	1.4(1.26~1.55)	<0.001	0.86(0.68~1.11)	0.249	1.42(1.15~1.77)	0.001	1.41(1.17~1.7)	<0.001	1.66(1.4~1.98)	<0.001
≥70	1.1(0.98~1.24)	0.118	0.47(0.34~0.66)	<0.001	0.67(0.52~0.87)	0.003	1.36(1.1~1.7)	0.005	1.63(1.33~1.99)	<0.001
**Diabetes drug**										
No(reference)	1		1		1		1		1	
Yes	0.78(0.68~0.9)	<0.001	0.76(0.55~1.01)	0.069	0.72(0.53~0.97)	0.033	0.98(0.78~1.24)	0.876	0.62(0.45~0.85)	0.003

**RA drug**										
No(reference)	1		1		1		1		1	
Yes	0.88(0.65~1.17)	0.382	0.61(0.15~1.75)	0.425	0.52(0.18~1.22)	0.176	0.98(0.59~1.61)	0.938	0.95(0.6~1.47)	0.814
**Asthma/COPD drug**										
No(reference)	1		1		1		1		1	
Yes	0.94(0.85~1.05)	0.271	0.76(0.53~1.07)	0.129	1.08(0.84~1.38)	0.543	0.91(0.75~1.1)	0.335	0.97(0.81~1.15)	0.712
**Antiepileptics**										
No(reference)	1		1		1		1		1	
Yes	0.94(0.77~1.15)	0.568	0.49(0.21~1)	0.077	1.19(0.75~1.84)	0.439	0.93(0.65~1.33)	0.697	0.99(0.71~1.37)	0.954
**Antiparkinson**										
No(reference)	1		1		1		1		1	
Yes	0.7(0.47~1.01)	0.067	0.59(0.09~2.03)	0.476	1.25(0.53~2.61)	0.581	0.58(0.28~1.13)	0.124	0.63(0.33~1.14)	0.143
**Psycholeptics**										
No(reference)	1		1		1		1		1	
Yes	0.86(0.79~0.94)	<0.001	1.03(0.8~1.3)	0.825	0.93(0.79~1.09)	0.392	0.84(0.69~1.01)	0.060	0.79(0.68~0.91)	0.001
**Psychoanaleptic**										
No(reference)	1		1		1		1		1	
Yes	1(0.91~1.1)	0.998	1.04(0.75~1.41)	0.815	0.95(0.77~1.17)	0.649	0.95(0.78~1.15)	0.586	1.07(0.92~1.25)	0.371
**Addictive_disorders**										

No(reference)	1		1		1		1		1	
Yes	0.77(0.53~1.12)	0.182	0.53(0.08~1.84)	0.392	1.09(0.25~3.39)	0.887	0.64(0.36~1.11)	0.122	1.04(0.56~1.89)	0.892
**Antineoplastic**										
No(reference)	1		1		1		1		1	
Yes	0.76(0.42~1.3)	0.331	0.6(0.03~3.15)	0.623	2.83(0.88~7.88)	0.057	0.56(0.2~1.39)	0.236	0.55(0.18~1.44)	0.251
**Calendar year**										
1996-2000(reference)	1		1		1		-	-	-	-
2000-2010	1.11(0.96~1.28)	0.173	1.17(0.91~1.51)	0.227	1.06(0.89~1.28)	0.500	-	-	-	-
2010-2020	3.67(3.19~4.23)	<0.001	-	-	-	-	-	-	-	-
**AUC**	0.511		0.5		0.5		0.526		0.528	
**Sensitivity(true positive rate)**	0.99		-		-		0.92		0.92	
**Specificity(true negative rate)**	0..03		-		-		0.13		0.13	
**Hosmer-Lemeshow test**	0.130		0.249		<0.001		0.602		0.006	
**Nagelkerke R^2**	0.124		0.028		0.046		0.041		0.038	

Reference group/category is set to 1.

-: No relevant data available.

Abbreviations: OR, Odds ratio; CI, Confidence interval; ACEIs, Angiotensin converting enzyme inhibitors; ARBs, Angiotensin II receptor blockers; BBs, Beta-blockers; CCBs, Calcium channel blockers; RA, Rheumatoid arthritis; COPD, Chronic obstructive pulmonary disease; AUC, area under the curve

Supplementary table 12. Logistic regression analysis of risk factors for discontinuation in patients on original antihypertensive monotherapy exceeding 360 days (1 year)

	Discontinuation		Cluster 1		Cluster 2		Cluster 3		Cluster 4	
**Anti-hypertensive drug class**	OR(95% CI)	p	OR(95% CI)	P	OR(95% CI)	p	OR(95% CI)	p	OR(95% CI)	p
BBs(reference)	1		1		1		1		1	
ACEIs	1.05(0.98~1.13)	0.185	1.08(0.92~1.26)	0.340	1.16(1~1.35)	0.055	0.85(0.74~0.97)	0.020	1.04(0.91~1.19)	0.576
ARBs	1.05(0.95~1.16)	0.320	1.18(0.96~1.46)	0.117	1.37(1.12~1.67)	0.002	0.83(0.69~1)	0.055	0.87(0.72~1.05)	0.150
CCBs	0.95(0.86~1.04)	0.267	1.22(0.91~1.65)	0.187	1.41(1.1~1.82)	0.007	0.72(0.6~0.86)	<0.001	0.82(0.7~0.97)	0.017
Thiazides	1.37(1.28~1.48)	<0.001	1.4(1.18~1.67)	<0.001	1.61(1.42~1.82)	<0.001	1.22(1.04~1.43)	0.016	1.18(1.03~1.34)	0.018
**Adherence**										
Low(reference)	1		1		1		1		1	
High	0.47(0.44~0.51)	<0.001	0.47(0.38~0.57)	<0.001	0.48(0.41~0.56)	<0.001	0.44(0.37~0.52)	<0.001	0.53(0.45~0.61)	<0.001
**Sex**										
Female(reference)	1		-	-	-	-	-	-	-	-
Male	1.06(1~1.11)	0.039	-	-	-	-	-	-	-	-
**Age**										
18-39(reference)	1		1		1		1		1	
40-69	0.5(0.45~0.54)	<0.001	0.64(0.52~0.79)	<0.001	0.44(0.37~0.53)	<0.001	0.54(0.45~0.65)	<0.001	0.46(0.39~0.54)	<0.001
≥70	0.72(0.65~0.8)	<0.001	1.19(0.93~1.54)	0.170	0.81(0.66~0.99)	0.045	0.67(0.54~0.82)	<0.001	0.5(0.42~0.61)	<0.001
**Diabetes drug**										
No(reference)	1		1		1		1		1	
Yes	1.08(0.96~1.21)	0.182	1.03(0.83~1.27)	0.807	1.11(0.9~1.38)	0.342	0.95(0.76~1.2)	0.685	1.24(0.93~1.66)	0.151

**RA drug**										
No(reference)	1		1		1		1		1	
Yes	0.99(0.76~1.29)	0.923	1.2(0.58~2.68)	0.636	1.57(0.86~3.05)	0.157	0.97(0.58~1.58)	0.890	0.77(0.49~1.2)	0.253
**Asthma/COPD drug**										
No(reference)	1		1		1		1		1	
Yes	1.11(1.01~1.23)	0.027	1.21(0.95~1.55)	0.124	1.15(0.94~1.41)	0.174	1.1(0.92~1.32)	0.302	1.02(0.86~1.21)	0.804
**Antiepileptics**										
No(reference)	1		1		1		1		1	
Yes	1.1(0.92~1.33)	0.284	1.85(1.13~3.19)	0.019	0.88(0.61~1.27)	0.474	1.12(0.79~1.6)	0.508	1.01(0.74~1.38)	0.960
**Antiparkinson**										
No(reference)	1		1		1		1		1	
Yes	1.75(1.25~2.5)	0.001	5.08(1.47~31.92)	0.029	1.59(0.81~3.34)	0.197	2.24(1.17~4.51)	0.018	1.2(0.69~2.12)	0.519
**Psycholeptics**										
No(reference)	1		1		1		1		1	
Yes	1.2(1.11~1.29)	<0.001	1.14(0.95~1.37)	0.163	1.06(0.94~1.2)	0.366	1.35(1.13~1.62)	0.001	1.33(1.15~1.52)	<0.001
**Psychoanaleptic**										
No(reference)	1		1		1		1		1	
Yes	1.09(1~1.19)	0.051	1.19(0.93~1.53)	0.174	1.25(1.07~1.48)	0.007	0.92(0.76~1.12)	0.422	1.01(0.87~1.17)	0.897
**Addictive_disorders**										

No(reference)	1		1		1		1		1	
Yes	1.26(0.9~1.78)	0.184	1.86(0.72~5.74)	0.230	1.73(0.6~6.2)	0.346	1.52(0.9~2.58)	0.116	0.76(0.41~1.37)	0.365
**Antineoplastic**										
No(reference)	1		1		1		1		1	
Yes	1.24(0.76~2.04)	0.390	1.5(0.44~6.85)	0.548	0.5(0.19~1.37)	0.167	1.91(0.81~4.83)	0.150	1.21(0.49~3.09)	0.675
**Calendar year**										
1996-2000(reference)	1		1		1		-	-	-	-
2000-2010	0.95(0.85~1.06)	0.329	0.96(0.8~1.15)	0.643	0.92(0.8~1.06)	0.262	-	-	-	-
2010-2020	0.49(0.44~0.54)	<0.001	-	-	-	-	-	-	-	-
**AUC**	0.592		0.5		0.499		0.568		0.553	
**Sensitivity(true positive rate)**	0.45		-		0		0.79		0.76	
**Specificity(true negative rate)**	0.73		-		1		0.35		0.35	
**Hosmer-Lemeshow test**	0.548		0.339		<0.001		0.207		0.053	
**Nagelkerke R^2**	0.083		0.050		0.065		0.056		0.047	

Reference group/category is set to 1.

-: No relevant data available.

Abbreviations: OR, Odds ratio; CI, Confidence interval; ACEIs, Angiotensin converting enzyme inhibitors; ARBs, Angiotensin II receptor blockers; BBs, Beta-blockers; CCBs, Calcium channel blockers; RA, Rheumatoid arthritis; COPD, Chronic obstructive pulmonary disease; AUC, area under the curve

Supplementary table 13. Logistic regression analysis of risk factors for switch in patients on original antihypertensive monotherapy exceeding 360 days (1 year)

	Switch		Cluster 1		Cluster 2		Cluster 3		Cluster 4	
**Anti-hypertensive drug class**	OR(95% CI)	p	OR(95% CI)	P	OR(95% CI)	p	OR(95% CI)	p	OR(95% CI)	p
BBs(reference)	1		1		1		1		1	
ACEIs	1.65(1.49~1.82)	<0.001	1.37(1.15~1.63)	<0.001	1.88(1.57~2.24)	<0.001	1.79(1.4~2.3)	<0.001	1.87(1.48~2.35)	<0.001
ARBs	1.99(1.75~2.25)	<0.001	2.26(1.82~2.81)	<0.001	2.65(2.14~3.28)	<0.001	1.56(1.12~2.16)	0.008	1.26(0.9~1.75)	0.173
CCBs	1.17(1.01~1.36)	0.039	1.12(0.8~1.56)	0.492	1.38(1.01~1.86)	0.036	1.27(0.92~1.74)	0.140	1.07(0.79~1.44)	0.666
Thiazides	2.07(1.88~2.27)	<0.001	1.89(1.57~2.27)	<0.001	1.95(1.68~2.27)	<0.001	3.04(2.36~3.94)	<0.001	2(1.6~2.51)	<0.001
**Adherence**										
Low(reference)	1		1		1		1		1	
High	1.28(1.15~1.43)	<0.001	1.36(1.12~1.67)	0.003	1.36(1.14~1.64)	<0.001	1.04(0.81~1.35)	0.762	1.23(0.94~1.63)	0.146
**Sex**										
Female(reference)	1		-	-	-	-	-	-	-	-
Male	1.23(1.14~1.32)	<0.001	-	-	-	-	-	-	-	-
**Age**										
18-39(reference)	1		1		1		1		1	
40-69	1.47(1.28~1.68)	<0.001	1.45(1.14~1.86)	0.003	1.68(1.33~2.14)	<0.001	1.22(0.9~1.71)	0.217	1.37(1.01~1.92)	0.054
≥70	1.57(1.35~1.83)	<0.001	1.23(0.93~1.64)	0.148	1.9(1.48~2.47)	<0.001	1.5(1.06~2.16)	0.026	1.62(1.14~2.32)	0.008
**Diabetes drug**										
No(reference)	1		1		1		1		1	
Yes	1.15(1~1.31)	0.048	1.21(0.96~1.51)	0.097	1.27(1.01~1.59)	0.041	1(0.69~1.42)	0.982	0.77(0.45~1.25)	0.319

**RA drug**										
No(reference)	1		1		1		1		1	
Yes	0.81(0.54~1.19)	0.309	0.6(0.2~1.45)	0.299	0.88(0.4~1.75)	0.734	0.51(0.15~1.24)	0.190	1.36(0.68~2.49)	0.349
**Asthma/COPD drug**										
No(reference)	1		1		1		1		1	
Yes	1.07(0.94~1.22)	0.278	0.95(0.73~1.22)	0.689	1.02(0.81~1.28)	0.847	1.08(0.81~1.42)	0.589	1.35(1.04~1.73)	0.021
**Antiepileptics**										
No(reference)	1		1		1		1		1	
Yes	0.9(0.69~1.16)	0.418	1.04(0.62~1.68)	0.862	0.92(0.56~1.44)	0.713	1.08(0.6~1.81)	0.788	0.6(0.29~1.08)	0.119
**Antiparkinson**										
No(reference)	1		1		1		1		1	
Yes	0.68(0.39~1.1)	0.140	1.15(0.41~2.78)	0.775	0.39(0.12~0.98)	0.077	0.44(0.07~1.45)	0.258	0.96(0.33~2.23)	0.930
**Psycholeptics**										
No(reference)	1		1		1		1		1	
Yes	1.03(0.94~1.14)	0.508	0.97(0.79~1.18)	0.754	0.97(0.83~1.12)	0.680	1(0.74~1.34)	0.979	1.34(1.07~1.67)	0.010
**Psychoanaleptic**										
No(reference)	1		1		1		1		1	
Yes	0.89(0.78~1.01)	0.065	1.09(0.84~1.42)	0.507	0.97(0.79~1.18)	0.737	0.76(0.54~1.06)	0.114	0.66(0.49~0.87)	0.005
**Addictive_disorders**										

No(reference)	1		1		1		1		1	
Yes	0.97(0.57~1.57)	0.909	0.55(0.13~1.67)	0.349	1.9(0.59~5.31)	0.242	1.17(0.48~2.45)	0.699	0.64(0.15~1.77)	0.454
**Antineoplastic**										
No(reference)	1		1		1		1		1	
Yes	0.73(0.32~1.46)	0.417	0.3(0.02~1.55)	0.251	0.26(0.01~1.29)	0.194	1.89(0.54~5.18)	0.258	1(0.16~3.53)	0.998
**Calendar year**										
1996-2000(reference)	1		1		1		-	-	-	-
2000-2010	0.9(0.79~1.02)	0.083	0.99(0.81~1.21)	0.927	0.82(0.7~0.96)	0.014	-	-	-	-
2010-2020	0.37(0.33~0.43)	<0.001	-	-	-	-	-	-	-	-
**AUC**	0.5		0.5		0.499		0.5		0.5	
**Sensitivity(true positive rate)**	-		-		1		-		-	
**Specificity(true negative rate)**	-		-		0		-		-	
**Hosmer-Lemeshow test**	0.047		0.056		0.012		0.120		0.063	
**Nagelkerke R^2**	0.070		0.038		0.051		0.034		0.032	

Reference group/category is set to 1.

-: No relevant data available.

Abbreviations: OR, Odds ratio; CI, Confidence interval; ACEIs, Angiotensin converting enzyme inhibitors; ARBs, Angiotensin II receptor blockers; BBs, Beta-blockers; CCBs, Calcium channel blockers; RA, Rheumatoid arthritis; COPD, Chronic obstructive pulmonary disease; AUC, area under the curve

Supplementary table 14. Logistic regression analysis of risk factors for add on in patients on original antihypertensive monotherapy exceeding 360 days (1 year)

	Add on		Cluster 1		Cluster 2		Cluster 3		Cluster 4	
**Anti-hypertensive drug class**	OR(95% CI)	p	OR(95% CI)	P	OR(95% CI)	p	OR(95% CI)	p	OR(95% CI)	p
BBs(reference)	1		1		1		1		1	
ACEIs	1.2(1.11~1.29)	<0.001	1.07(0.92~1.24)	0.370	1.12(0.97~1.29)	0.128	1.56(1.32~1.83)	<0.001	1.3(1.1~1.52)	0.001
ARBs	1.22(1.1~1.35)	<0.001	1.16(0.95~1.41)	0.153	1.21(1~1.46)	0.049	1.46(1.17~1.81)	<0.001	1.3(1.05~1.61)	0.015
CCBs	0.84(0.75~0.94)	0.002	0.85(0.64~1.11)	0.230	0.93(0.73~1.17)	0.537	1.04(0.84~1.28)	0.738	0.84(0.68~1.02)	0.086
Thiazides	1.49(1.38~1.6)	<0.001	1.28(1.09~1.5)	0.003	1.13(1~1.27)	0.042	2.49(2.08~2.98)	<0.001	1.82(1.57~2.12)	<0.001
**Adherence**										
Low(reference)	1		1		1		1		1	
High	1.71(1.57~1.87)	<0.001	1.83(1.55~2.17)	<0.001	1.89(1.64~2.18)	<0.001	1.44(1.19~1.74)	<0.001	1.48(1.22~1.8)	<0.001
**Sex**										
Female(reference)	1		-	-	-	-	-	-	-	-
Male	1.14(1.08~1.2)	<0.001	-	-	-	-	-	-	-	-
**Age**										
18-39(reference)	1		1		1		1		1	
40-69	2.41(2.17~2.68)	<0.001	2.48(2.02~3.06)	<0.001	2.68(2.25~3.21)	<0.001	1.98(1.56~2.53)	<0.001	2.22(1.76~2.83)	<0.001
≥70	2.46(2.19~2.77)	<0.001	2.13(1.68~2.71)	<0.001	3.21(2.64~3.92)	<0.001	1.93(1.48~2.54)	<0.001	2.25(1.74~2.93)	<0.001
**Diabetes drug**										
No(reference)	1		1		1		1		1	
Yes	1.33(1.19~1.49)	<0.001	1.54(1.26~1.89)	<0.001	1.38(1.13~1.69)	0.002	0.99(0.77~1.28)	0.961	1.42(1.03~1.94)	0.028

**RA drug**										
No(reference)	1		1		1		1		1	
Yes	1.14(0.86~1.49)	0.366	1.08(0.53~2.18)	0.838	0.84(0.47~1.46)	0.538	0.96(0.54~1.65)	0.895	1.68(1.05~2.64)	0.027
**Asthma/COPD drug**										
No(reference)	1		1		1		1		1	
Yes	1.04(0.94~1.15)	0.456	1.05(0.84~1.3)	0.685	0.97(0.8~1.17)	0.758	1.08(0.88~1.32)	0.453	1.13(0.93~1.37)	0.202
**Antiepileptics**										
No(reference)	1		1		1		1		1	
Yes	0.89(0.73~1.08)	0.248	0.89(0.58~1.36)	0.589	1.11(0.77~1.57)	0.577	0.8(0.52~1.21)	0.315	0.78(0.52~1.14)	0.206
**Antiparkinson**										
No(reference)	1		1		1		1		1	
Yes	0.56(0.38~0.81)	0.003	0.46(0.18~1.08)	0.088	0.33(0.15~0.65)	0.002	0.4(0.14~0.95)	0.060	1.28(0.67~2.31)	0.437
**Psycholeptics**										
No(reference)	1		1		1		1		1	
Yes	0.99(0.92~1.07)	0.793	0.92(0.78~1.1)	0.366	1.02(0.91~1.15)	0.705	0.86(0.7~1.07)	0.179	1.03(0.87~1.21)	0.723
**Psychoanaleptic**										
No(reference)	1		1		1		1		1	
Yes	0.86(0.78~0.94)	0.002	0.81(0.65~1.02)	0.077	0.9(0.77~1.05)	0.168	1(0.8~1.24)	0.973	0.76(0.63~0.92)	0.004
**Addictive_disorders**										

No(reference)	1		1		1		1		1	
Yes	1.03(0.71~1.48)	0.877	1(0.41~2.41)	0.994	0.78(0.26~2.1)	0.628	1.13(0.62~1.98)	0.678	1.02(0.49~1.97)	0.953
**Antineoplastic**										
No(reference)	1		1		1		1		1	
Yes	0.85(0.5~1.41)	0.537	0.66(0.2~2)	0.469	0.7(0.24~1.89)	0.495	0.83(0.27~2.15)	0.720	1.36(0.47~3.5)	0.545
**Calendar year**										
1996-2000(reference)	1		1		1		-	-	-	-
2000-2010	1.01(0.91~1.12)	0.871	0.99(0.83~1.17)	0.884	1.04(0.92~1.19)	0.520	-	-	-	-
2010-2020	0.4(0.36~0.45)	<0.001	-	-	-	-	-	-	-	-
**AUC**	0.563		0.552		0.533		0.5		0.5	
**Sensitivity(true positive rate)**	0.88		0.44		0.81		-		-	
**Specificity(true negative rate)**	0.24		0.67		0.25		-		-	
**Hosmer-Lemeshow test**	<0.001		0.020		0.179		0.222		0.119	
**Nagelkerke R^2**	0.100		0.058		0.060		0.049		0.050	

Reference group/category is set to 1.

-: No relevant data available.

Abbreviations: OR, Odds ratio; CI, Confidence interval; ACEIs, Angiotensin converting enzyme inhibitors; ARBs, Angiotensin II receptor blockers; BBs, Beta-blockers; CCBs, Calcium channel blockers; RA, Rheumatoid arthritis; COPD, Chronic obstructive pulmonary disease; AUC, area under the curve

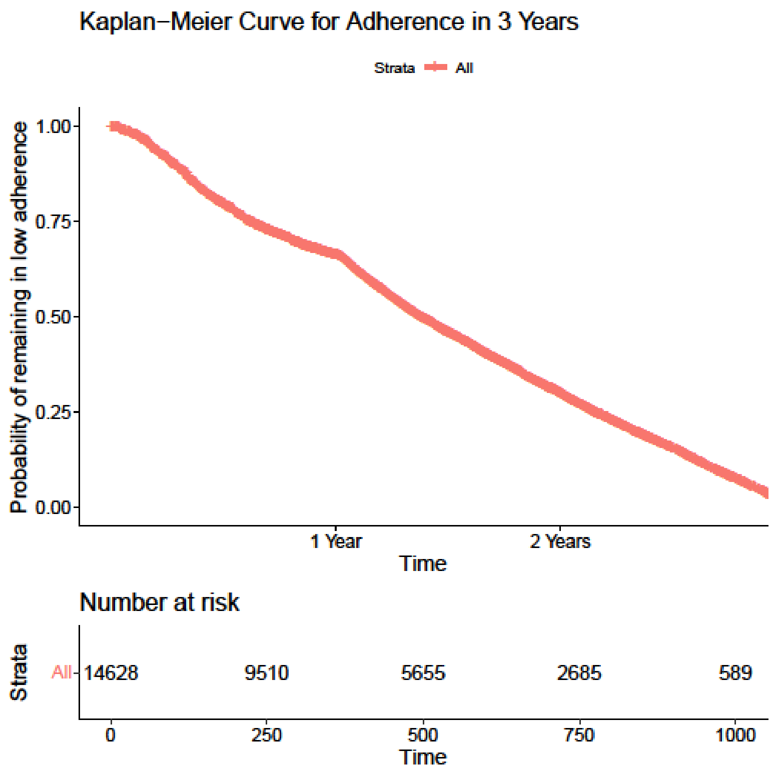

A. Before IPW (low adherence as reference)

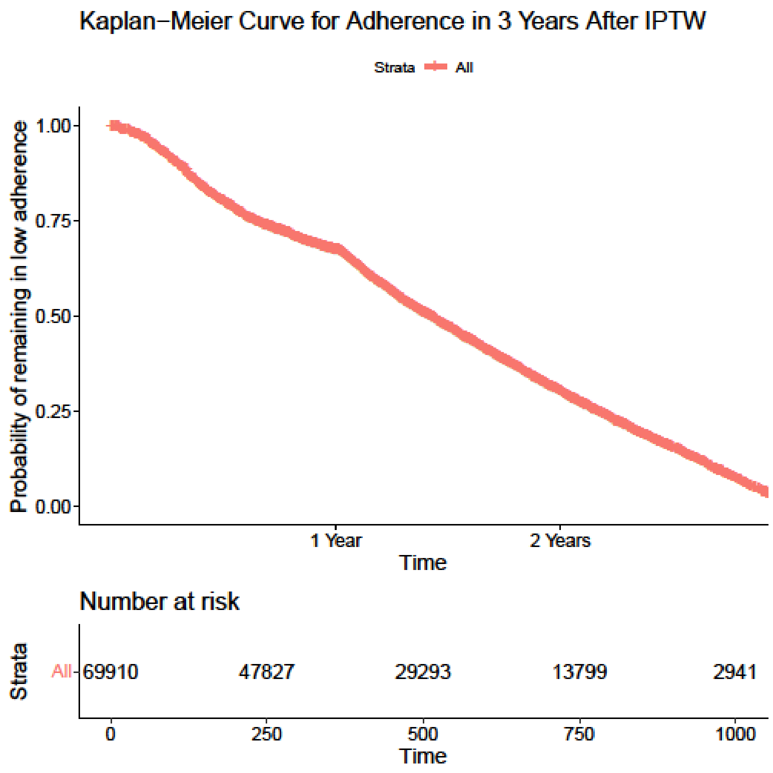

B. After IPW (low adherence as reference)

Supplementary figure 1. Kaplan-Meier curve for adherence (14628 patients on original antihypertensive monotherapy ≤1080 days in 27783 patients with all drug records exceed 1080 days). A. Before IPW (low adherence as reference), B. After IPW (low adherence as reference).

Abbreviations: ACEIs, Angiotensin converting enzyme inhibitors; ARBs, Angiotensin II receptor blockers; BBs, Beta-blockers; CCBs, Calcium channel blockers; IPW, inverse probability weighting

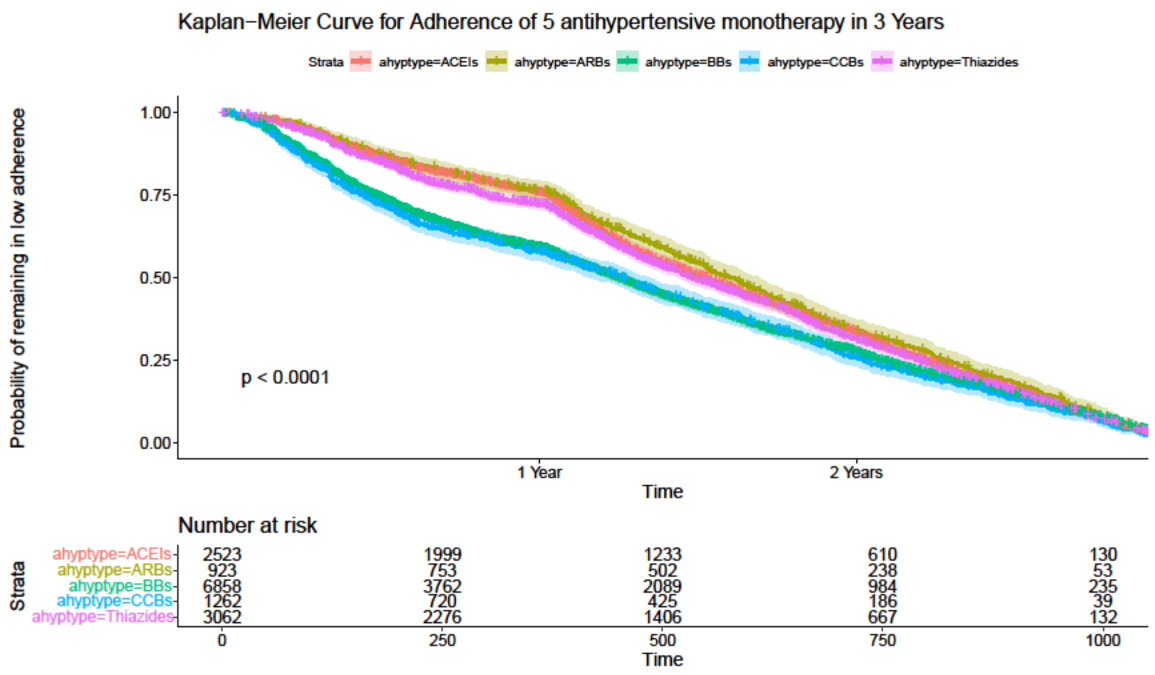

A. Before IPW (low adherence as reference)

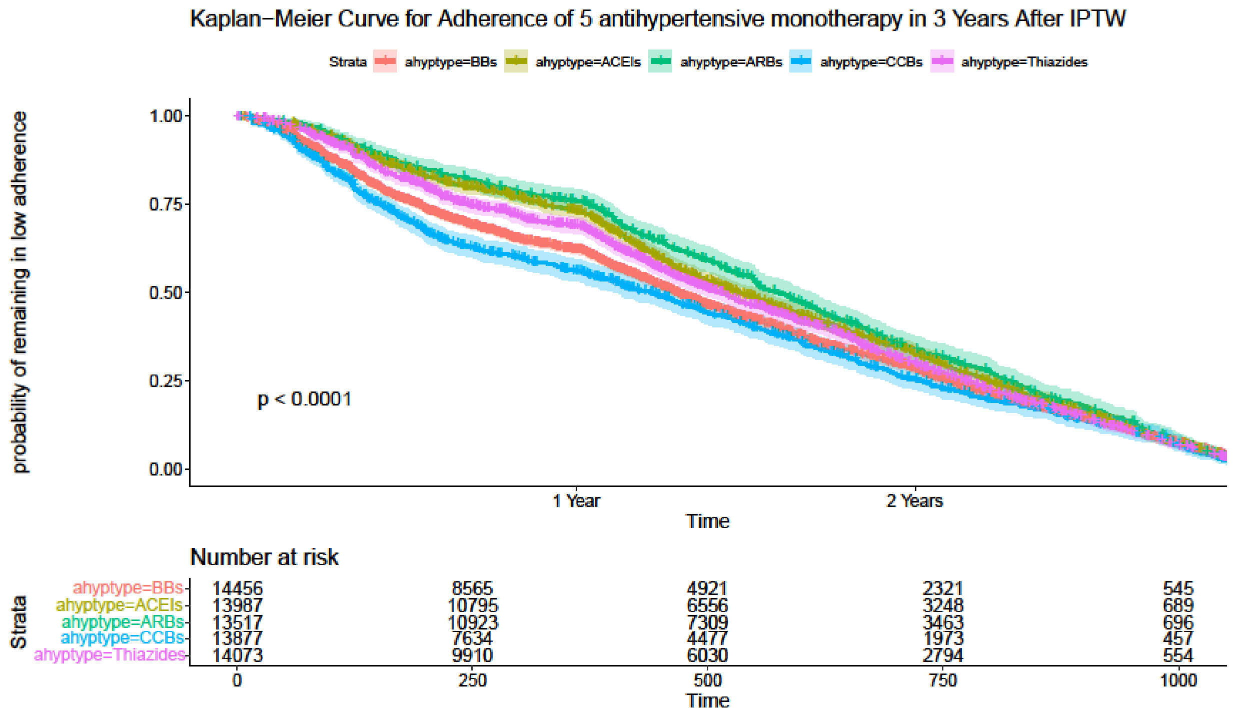

B. After IPW (low adherence as reference)

Supplementary figure 2. Kaplan-Meier curve for adherence of 5 classes of antihypertensive drug monotherapy (14628 patients on original antihypertensive monotherapy ≤1080 days in 27783 patients with all drug records exceed 1080 days. A. Before IPW (low adherence as reference), B. After IPW (low adherence as reference)

Abbreviations: ACEIs, Angiotensin converting enzyme inhibitors; ARBs, Angiotensin II receptor blockers; BBs, Beta-blockers; CCBs, Calcium channel blockers; IPW, inverse probability weighting
